# Enhancing Cell Penetration Efficiency of Cyclic Oligoarginines Using Rigid Scaffolds

**DOI:** 10.3390/pharmaceutics15061736

**Published:** 2023-06-14

**Authors:** Csaba Bató, Ildikó Szabó, Zoltán Bánóczi

**Affiliations:** 1 Department of Organic Chemistry, Institute of Chemistry, Eötvös Loránd University, 1117 Budapest, Hungary; bs.csabi@gmail.com; 2 ELKH-ELTE Research Group of Peptide Chemistry, 1117 Budapest, Hungary; ildiko.szabo@ttk.elte.hu

**Keywords:** cell-penetrating peptide, cyclic peptide, transport molecule, enhancing internalization

## Abstract

Delivering therapeutic agents into cells has always been a major challenge. In recent years, cyclization emerged as a tool for designing CPPs to increase their internalization and stability. Cyclic ring(s) can protect the peptide from enzymatic degradation, so cyclic peptides remain intact. Therefore they can be good carrier molecules. In this work, the preparation and investigation of efficient cyclic CPPs are described. Different oligoarginines were designed to conjugate with rigid aromatic scaffolds or form disulfide bonds. The reaction between the scaffolds and the peptides forms stable thioether bonds, constraining the peptide into a cyclic structure. The constructs presented very efficient internalization on cancerous cell lines. Our peptides use more than one endocytic pathway for cellular uptake. In this way, short peptides, which can compete with the penetration of well-known CPPs such as octaarginine (Arg_8_), may be synthesized through cyclization.

## 1. Introduction

Delivering therapeutic agents into cells is always a major challenge; thus, the need for a reliable transport system is high. Cell-penetrating peptides (CPP) can be promising transport molecules because they are capable of delivering proteins [1], drugs [2,3,4,5,6,7], and other therapeutic peptides [8,9,10] into the cells.

The first CPP, the Tat peptide derived from the human immunodeficiency virus HIV-1, was described in 1988 [11]. In parallel, the cell-penetration properties of a peptide, penetratin, from the Drosophila Antenapedia protein were revealed, too [12]. These discoveries were a milestone in the research of cell-penetrating peptides. Both peptides are positively charged because of the arginine residues and show remarkable internalization properties. Later, it turned out that the positively charged side chains (guanidinium group) of arginines are the reason for the high internalization [13,14]. This led to the synthesis of oligoarginines, such as octaarginine (Arg_8_), as cell-penetrating peptides [14].

These peptides with increased arginine content are effective CPPs, but their activity can be improved with different modifications [15]. For example, their N-terminus can be modified with a hydrophobic moiety [16,17]. Earlier, we measured that the Dabcyl group (4-((4-(dimethylamino)phenyl)azo)benzoic acid), a commonly used quencher in FRET pairs [18,19], is an excellent uptake enhancer when coupled to oligoarginines. Drastically increased internalization of its hexaarginine derivative was detected [20]. It had higher internalization than the octaarginine. Although the Dabcyl-modified tetraarginine was not so effective; it showed marked direct penetration. Thus, we modified its sequence with Trp, an aromatic amino acid [21], because Trp moieties may increase the penetration of oligoarginines [21,22,23]. However, the Trp alone did not enhance the cellular uptake of tetraarginine, but it was very efficient in combination with the Dabcyl group [24]. Tetraarginine with a Trp and a Dabcyl group at its N-terminus was very efficient. In addition to this, aromatic moieties in the backbone could improve the internalization of tetraarginine, an inefficient cell-penetrating peptide, too [25,26].

Cyclization of a peptide may increase the biological activity and/or the resistance against enzymatic degradation. Cyclic peptides are promising candidates for fighting cancerous diseases. Countless novel cyclic peptides are derived from nature exhibiting diverse biological activities [27]. For example, two bicyclic octapeptides, Moroidin and Celogentins A–K, were isolated from the seeds of Celosia argentea. They have inhibitory activity against tubulin polymerization and prevent eukaryotic cell division [28] or antimitotic activity [29].

The cyclic peptides have constrained conformation because of less flexibility caused by cyclization. The predominated conformation may improve its binding properties [30]. Cyclization may enhance the interaction area and, thus, the activity of a peptide [31]. Many times these peptides have enhanced serum stability, which results in increased resistance against protease degradation [32]. The enhanced stability means more time for interaction with the cell membrane and, therefore, may result in better internalization [33]. Additionally, many bicyclic peptides were reported with strong cellular uptake and applicability for cellular delivery. These peptides are usually small in size, which can lead to rapid and deep tissue penetration. With these advantages, novel transport molecules can be designed that can bring therapeutic agents into the cells.

In this work, we investigated the effect of cyclization on the internalization of different oligoarginines. We used disulfide bonds or rigid aromatic scaffolds to prepare cyclic or bicyclic peptides with modification of the N-terminus with the Dabcyl group. We assume that the Dabcyl group and cyclization together will increase the internalization of short peptides.

## 2. Materials and Methods

### 2.1. Synthesis of Peptides and Their Conjugates

Manually Fmoc/*^t^*Bu solid phase peptide synthesis (SPPS) was applied on Rink amide MBHA resin to synthesize the peptides. The arginine was protected by 2,2,4,6,7-pentamethyldihydrobenzofuran-5-sulfonyl (Pbf), cysteine by triphenyl methyl (Trt), and lysine by the *tert*-butyloxycarbonyl (Boc) group. The temporally α-amino protection, Fmoc group, was removed by the solution of 2% piperidine and 2% 1,8-diazabicycloundec-7-ene (DBU) in dimethyl formamide (DMF) (2 + 2 + 5 + 10 min). For the coupling, 3 eq molar excess of N,N′-diisopropylcarbodiimide (DIC) and ethyl cyano(hydroxyimino)acetate (Oxima Pure) as coupling reagents and amino acid derivative were used. They were dissolved in DMF and were applied for 60 min at RT. Then, the resin was washed with DMF (2 × 1 min) and dichloromethane (DCM) (3 × 1 min). The efficacy of the coupling was tested by a ninhydrin probe. In the case of a negative test, this process was repeated until the whole peptide was built up. In the last step, the N-terminal modification was done; 4-((4-(dimethylamino)phenyl)azo)benzoic acid (Dabcyl-OH) using Oxima Pure/DIC or acetyl group using acetic anhydride was coupled to the peptide. The cleavage of the peptides was done using 5 mL TFA containing 0.365 g phenol, 0.25 mL distilled water, 0.25 mL thioanisole, and 0.125 mL TIS as scavengers. The cleaved peptides were precipitated and washed twice by dry diethyl-ether, dissolved in 10% acetic acid, lyophilized, and purified by semi-preparative HPLC. The peptides were characterized by analytical RP-HPLC and ESI-MS. The purity of the compounds was higher than 90%.

The cyclization took place in a 50–50% tris(hydroxymethyl)aminomethane (Tris) buffer/acetonitrile (ACN) solution in RT. The concentration of the solution for the peptides was 1 mM. The reaction took place for two days. The crude products were purified by semi-preparative HPLC. The chemical characterization of the purified compounds was done by analytical RP-HPLC and ESI-MS. The purity of the compounds was higher than 90%.

Every peptide has Lys residue to attach the Cf or succinylated daunomycin (DauSuc). After the successful cyclization, the peptides were reacted with Cf or DauSuc. The reaction took place in the solution phase where the peptides reacted with 1 eq of dye or drug, 1 eq of DIC, and Oxima Pure as coupling reagents in the presence of 3 eq of N,N-diisopropylethylamine (DIEA) that were used in DMF. The reaction was stirred overnight. Eluent A was added to the reaction mixture in the appropriate amount, and it was purified by RP-HPLC.

### 2.2. Flow Cytometry

#### EBC-1

The internalization study of fluorophore-labeled peptides was performed on 24-well plates with 10^5^ EBC-1 human lung squamous cancer cells (CRL-5889TM) per well. After 24 h incubation at 37 °C, the cells were treated with a serum-free medium as a negative control and with peptides in different concentrations (1, 5, 10 μM). After the incubation, 100 μL trypsin (0.25%) was used for 5–10 min to remove the excess peptides from the membranes and to detach the cells from the plates. By adding 800 µL of HPMI buffer (glucose, NaHCO_3_, NaCl, HEPES, KCl, MgCl_2_, CaCl_2_, Na_2_HPO_4_ × 2 H_2_O) containing 10% fetal bovine serum (FBS), the trypsin activity was stopped, and the cells were transferred to tubes for measurement. The cells were centrifuged at 216× *g* at 4 °C, and the supernatant was disposed of. The resuspended cells (in 250 μL HPMI) were used to measure their fluorescence intensity by flow cytometer (BDLSR II, BD Bioscience, San Jose, CA, USA). Data were analyzed with FACSDiVa 5.0 software (BD Bioscience, San Jose, CA, USA).

In order to study the effect of inhibitors, the peptide conjugates were administered at 5 μM when the cells were pretreated by the inhibitors for 30 min. The cells were then kept at 37 °C for 90 min. Macropinocytosis was inhibited using 5-(N-ethyl-N-isopropyl)amiloride (EIPA) [34], clathrin-mediated endocytosis with chlorpromazine (CPZ) [35], colchicine (Col) to assess the role of microtubules [36], and methyl-β-cyclodextrin (CyD) for the inhibition of caveolae/lipid-raft-mediated endocytosis [37].

### 2.3. Analysis of In Vitro Cytostatic Activity of Conjugates

The 5 × 10^3^ EBC-1 cells per well were used in the in vitro measurement on 96-well plates. After 24 h incubation at 37 °C, the cells were treated by the compounds in a final volume of 200 μL for 3 h. The cytostatic activity was measured at concentrations between 0.000128 and 100 μM. A serum-free medium was used to treat control cells for 3 h at 37 °C. Then the compounds were washed out using a serum-free medium twice. Cells were subsequently cultured for an additional 72 h in a medium containing serum. The IC_50_ values were then determined using the Alamar-blue (Resazurim) assay. Hence, 22.5 μL of Alamar-blue solution was added to each well (0.15 mg/mL, PBS pH = 7 was used as a solvent). After 4 h of incubation, the fluorescence was measured using a Synergy H4 multimode microplate reader (BioTek, Winooski, VT, USA) at λ = 530/30 nm and 610/10 nm. To measure the percentage of cytostasis, the following equation was used for calculation: Cytostatic effect (%) = [1 − (OD_treated_/OD_control_)] × 100; where OD_treated_ and OD_control_ correspond to the optical densities of the treated and untreated cells, respectively. Four parallel measurements were conducted in two independent experiments. The dose-response curves were used to calculate the 50% inhibitory concentration (IC_50_) values. The curves were defined using MicrocalTM Origin (version 8.0, OriginLab, Northampton, MA, USA) software: cytostasis was plotted as a function of concentration, fitted to a sigmoidal curve, and based on this curve, the IC_50_ value was determined. IC_50_ represented the concentration of a compound required for 50% inhibition in vitro and expressed as micromolar units.

### 2.4. Statistical Analysis

The mean value standard deviation is used to represent the outcomes of the uptake studies. The statistical analysis was made by the use of the Student’s *t*-test. Results were considered statistically significant if their *p*-values were less than 0.05.

## 3. Results

### 3.1. Synthesis of Peptides

**Figure 1:**
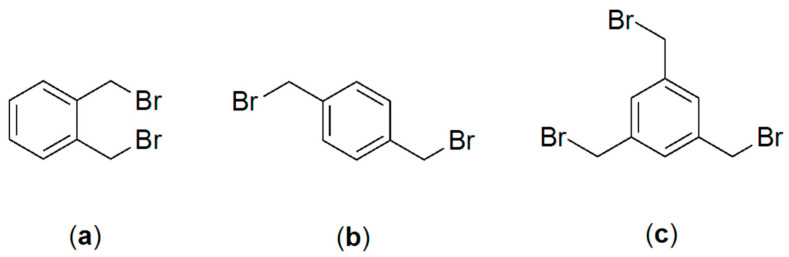
Structure of the used rigid scaffolds: (**a**) 1,2-bis(bromomethyl)benzene (orto BBMB), (**b**) 1,4-bis(bromomethyl)benzene (para BBMB), (**c**) 1,3,5-tris(bromomethyl)benzene (TBMB).

**Scheme 1:**
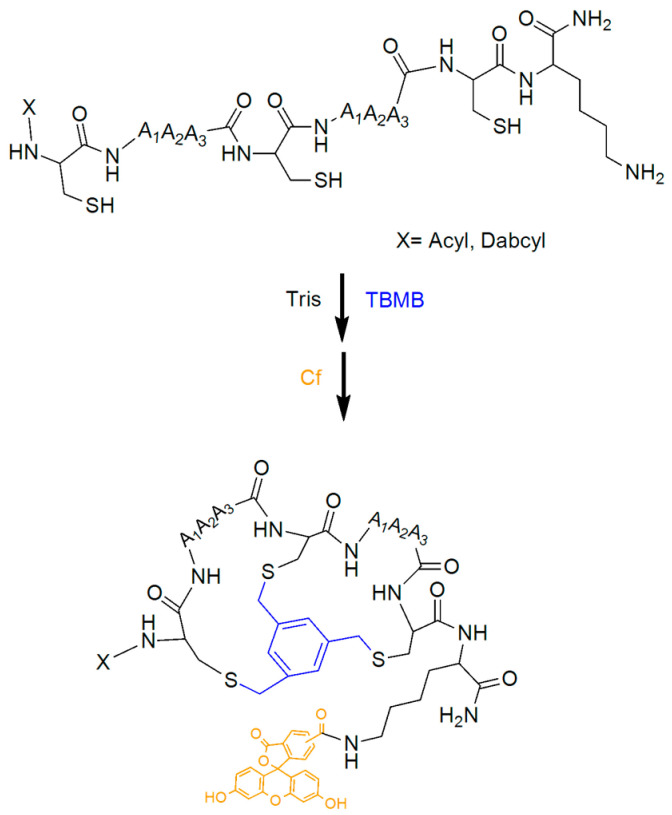
Cyclization reaction between peptides and TBMB scaffold.

**Scheme 2:**
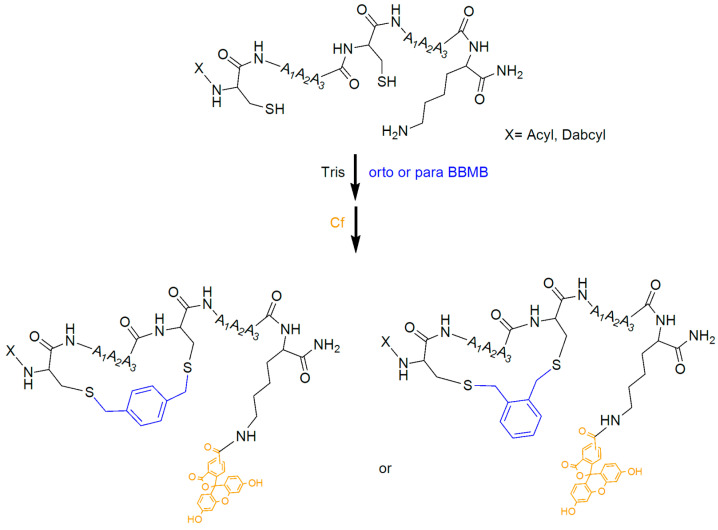
Cyclization reaction between peptides and BBMB scaffolds.

**Scheme 3:**
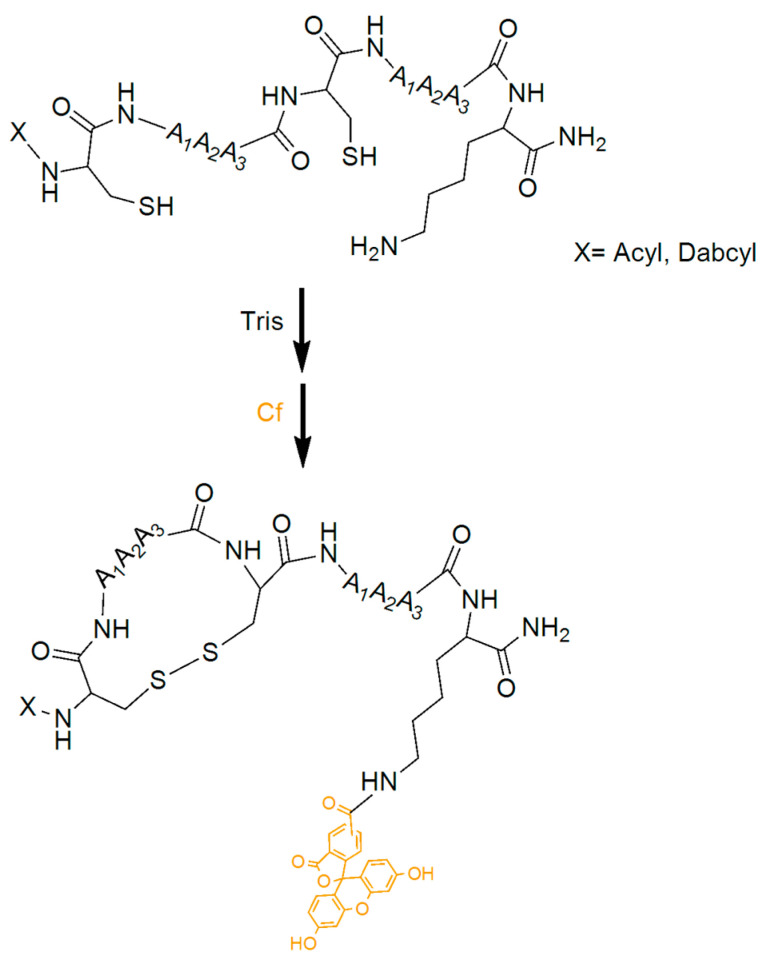
Synthesis of a cyclic peptide with a disulfide bond.

In this work, monocyclic and bicyclic peptides were designed to study the effect of cyclization on oligoarginines internalization. Therefore, all peptides contain cysteine residues that can react with each other to form disulfide bonds or with the bromomethyl groups of different bromomethyl–benzene derivatives (Figure 1, Scheme 1 and Scheme 2) to form thioether bonds. To examine the effect of cyclization and aromatic ring in the cycle, linear and cyclic peptides with disulfide bonds (Scheme 3) were prepared. Every sequence contains a tetraglycine unit as a model cargo peptide. A manual Fmoc/^t^Bu strategy was used to synthesize the peptides. For the N-terminal modification, Dabcyl and acetyl groups were used. The amino acids and Dabcyl group were coupled using DIC and Oxima Pure. Acetylation was done by acetic anhydride and DIEA. The cyclization in all cases was done in the solution phase using a Tris buffer pH = 8.6 and ACN. To study the uptake of peptides, they were labeled by the fluorescent dye, 5(6)-carboxyfluorescein (Cf), at their C-terminal on the ε-amino group of Lys in a solution using DIC and Oxima Pure. In the synthesis of conjugates with an antitumor drug (succinylated daunomycin (DauSuc)), the drug molecule was attached to the Lys side chain in a solution using DIC and Oxima Pure coupling reagents; (Scheme 1, Scheme 2 and Scheme 3).

The chemical characterization of peptides and conjugates was carried out by ESI-MS and analytical RP-HPLC (Table 1; analytical RP-HPLC chromatograms and MS spectra are in the Supplementary Materials, Figures S1–S50).

### 3.2. Cellular Uptake

**Figure 2:**
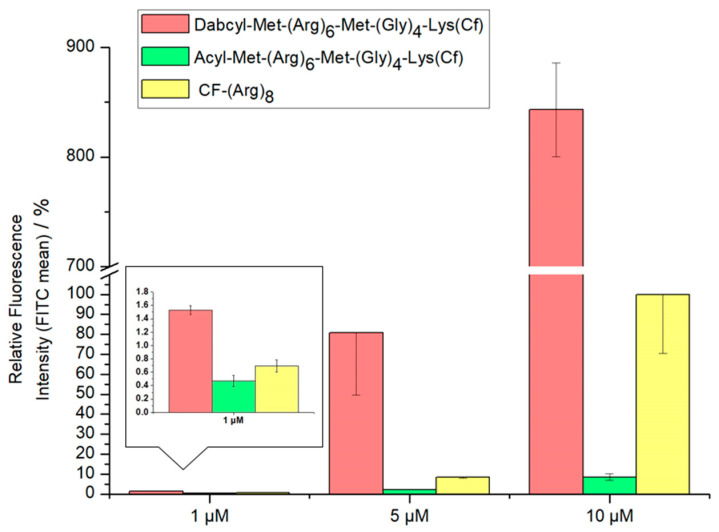
Cellular uptake of linear peptides on EBC-1 cells. Peptides were applied in 1, 5, and 10 μM concentrations in 90 min treatment. Flow cytometry was used to determine the fluorescence intensity of the cells. The fluorescence intensity is relative to Cf-R_8_ at 10 μM (100%). Data represent the mean ± standard deviation (SD).

The internalization of peptides was measured by flow cytometry on EBC-1 cells. The carboxyfluorescein-labeled peptides were studied at 1, 5, and 10 μM concentrations. The cells were treated for 90 min at 37 °C. The peptides showed concentration-dependent internalization. The uptake of Cf-Arg_8_ and Dabcyl-Met-(Arg)_6_-Met-(Gly)_4_-Lys(Cf) linear control peptides increased exponentially, while the Acyl-Met-(Arg)_6_-Met-(Gly)_4_-Lys(Cf) linear control peptide showed linearly increased uptake with the enhancing concentration (Figure 2). The Dabcyl-containing linear peptide had 8–10 fold better internalization than the octaarginine.

**Figure 3:**
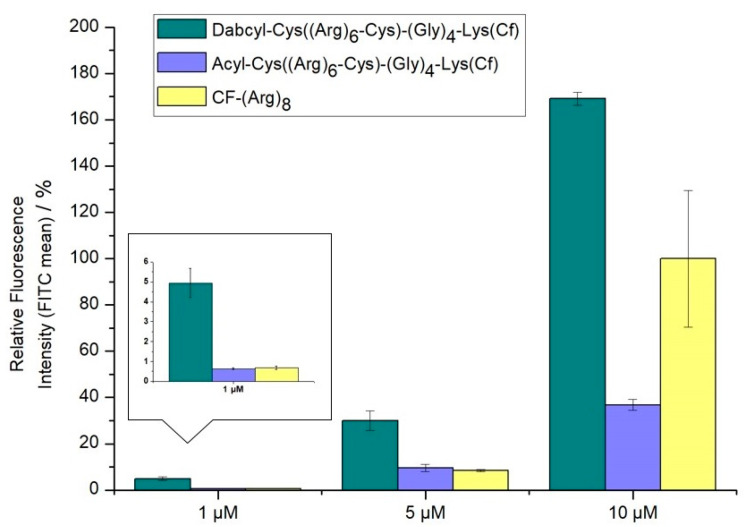
Cellular uptake of cyclic peptides with disulfide bond on EBC-1 cells. Peptides were applied in 1, 5, and 10 μM concentrations in 90 min treatment. Flow cytometry was used to determine the fluorescence intensity of the cells. The fluorescence intensity is relative to Cf-R_8_ at 10 μM (100%). Data represent the mean ± standard deviation (SD).

In the next step, the effect of cyclization on internalization was examined (Figure 3). The cyclic peptide was formed via a disulfide bond. The cyclic peptide (Acyl-Cys((Arg)_6_-Cys)-(Gly)_4_-Lys(Cf)) showed better cellular uptake than the linear peptide (Acyl-Met-(Arg)_6_-Met-(Gly)_4_-Lys(Cf)), and it had the same or almost half efficiency than the octaarginine at 5 and 10 µM concentrations, respectively. The Dabcyl group had internalization-enhancing ability in the case of this cyclic peptide (Dabcyl-Cys((Arg)_6_-Cys)-(Gly)_4_-Lys(Cf)), too. This derivative had 1.5–2 fold higher cellular uptake than the octaarginine.

**Figure 4:**
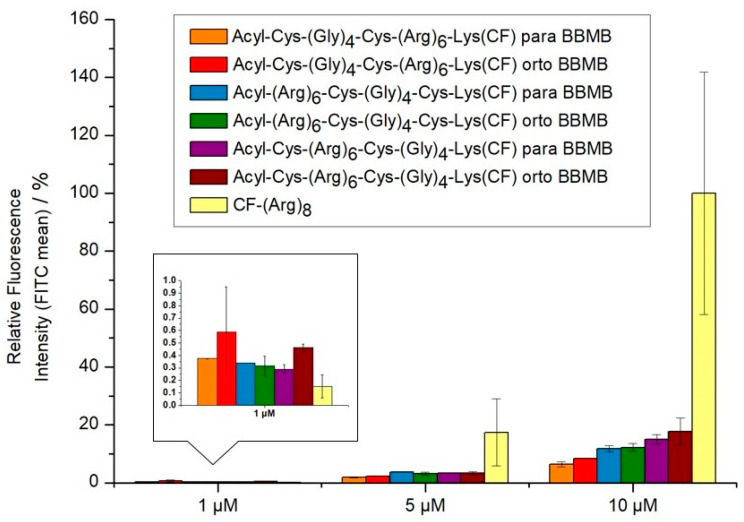
Cellular uptake of cyclic peptides with benzene ring on EBC-1 cells. Peptides were applied in 1, 5, and 10 μM concentrations in 90 min treatment. Flow cytometry was used to determine the fluorescence intensity of the cells. The fluorescence intensity is relative to Cf-R_8_ at 10 μM (100%). Data represent the mean ± standard deviation (SD).

Benzene derivatives (di- and tribromomethylene modified, Figure 1) are commonly used as a scaffold to synthesize cyclic or bicyclic peptides forming thioether bonds between the thiol groups in the peptide and the benzene ring. Using this kind of cyclization results in a cyclic compound with additional aromatic moiety. Two different scaffolds were used to prepare two cyclic variants of our model peptides, which are different only in the size of the ring. In these derivatives, the position of hexaarginine as cell-penetrating moiety and tetraglycine a model peptide sequence as cargo is changed; the hexaarginine is in (Acyl-Cys-(Arg)_6_-Cys-(Gly)_4_-Lys(Cf) para BBMB) or out (Acyl-Cys-(Gly)_4_-Cys-(Arg)_6_-Lys(Cf) para BBMB) of the ring; at the N- (Acyl-(Arg)_6_-Cys-(Gly)_4_-Cys-Lys(Cf) para BBMB) or C-terminus (Acyl-Cys-(Gly)_4_-Cys-(Arg)_6_-Lys(Cf) para BBMB). The cyclic peptides had very similar internalization, which did not show any dependence on the arrangement of peptides (Figure 4). The size of the ring (orto (Acyl-Cys-(Arg)_6_-Cys-(Gly)_4_-Lys(Cf) orto BBMB)) or para position (Acyl-Cys-(Arg)_6_-Cys-(Gly)_4_-Lys(Cf) para BBMB) of the thioether bonds) did not have an effect on cellular uptake either. In comparison with disulfide cyclic peptide (Acyl-Cys((Arg)_6_-Cys)-(Gly)_4_-Lys(Cf)), these peptides were less effective. Their internalization was half of Acyl-Cys((Arg)_6_-Cys)-(Gly)_4_-Lys(Cf). However, they were more active than the octaarginine at 1 µM concentration.

**Figure 5:**
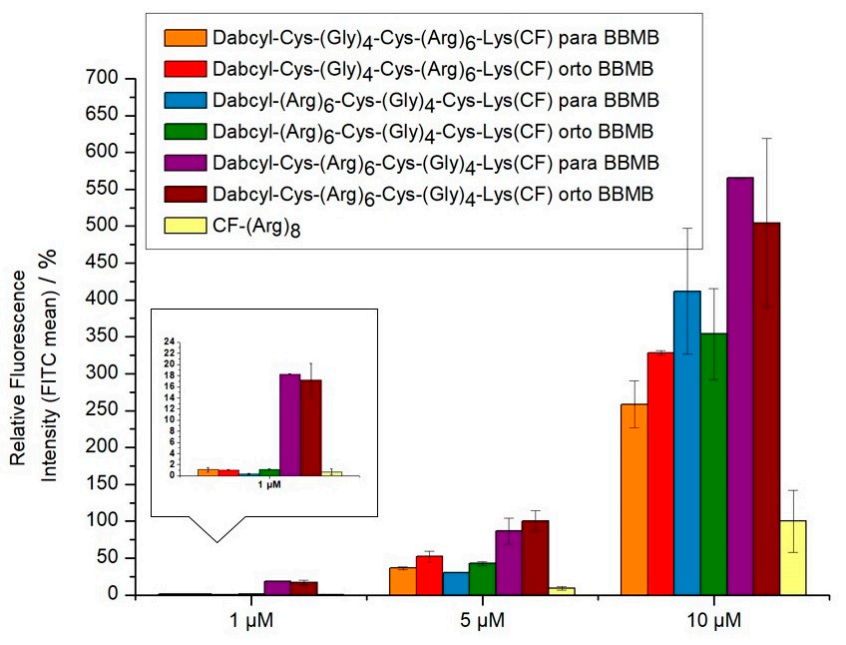
Cellular uptake of cyclic peptides with a benzene ring and Dabcyl group on EBC-1 cells. Peptides were applied in 1, 5, and 10 μM concentrations in 90 min treatment. Flow cytometry was used to determine the fluorescence intensity of the cells. The fluorescence intensity is relative to Cf-R_8_ at 10 μM (100%). Data represent the mean ± standard deviation (SD).

When the Dabcyl group was introduced into the N-terminus, these cyclic peptides had enhanced cellular uptake. All peptides had higher internalization at all concentrations than the octaarginine (Figure 5). There is no significant difference in the internalization of orto (e.g., Dabcyl-Cys-(Arg)_6_-Cys-(Gly)_4_-Lys(Cf) orto BBMB) and para (e.g., Dabcyl-Cys-(Arg)_6_-Cys-(Gly)_4_-Lys(Cf) para BBMB) peptides. The two best peptides were Dabcyl-Cys-(Arg)_6_-Cys-(Gly)_4_-Lys(Cf) para and orto BBMB, in which the hexaarginine moiety was in the cycle. Their internalization was 5-fold better than octaarginine and was outstanding in low concentration as well. The peptide Dabcyl-Cys-(Arg)_6_-Cys-(Gly)_4_-Lys(Cf) para BBMB was the only one that showed toxicity in 10 µM concentration.

**Figure 6:**
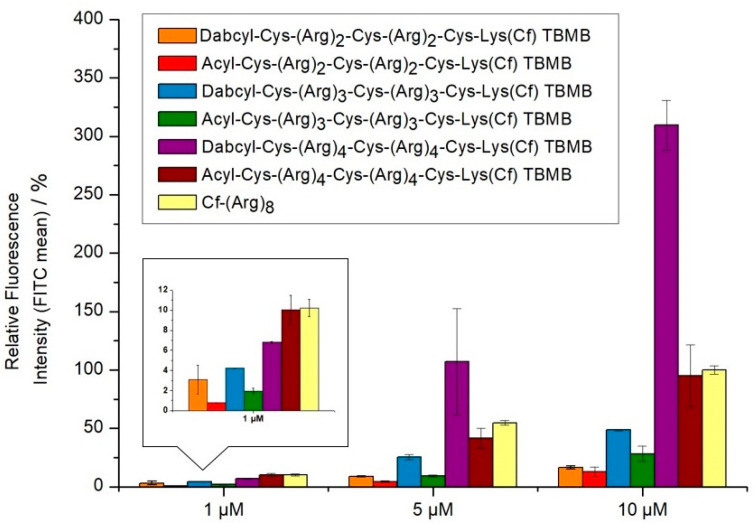
Cellular uptake of bicycle peptides on EBC-1 cells. Peptides were applied in 1, 5, and 10 μM concentrations in 90 min treatment. Flow cytometry was used to determine the fluorescence intensity of the cells. The fluorescence intensity is relative to Cf-R_8_ at 10 μM (100%). Data represent the mean ± standard deviation (SD).

In the case of bicyclic peptides, the arginine residues were introduced into both cycles in the same number. There was no difference between the efficacy of tetra- and hexaarginine derivatives (Acyl-Cys-(Arg)_2_-Cys-(Arg)_2_-Cys-Lys(Cf) TBMB and Acyl-Cys-(Arg)_3_-Cys-(Arg)_3_-Cys-Lys(Cf) TBMB), while the bicyclic octaarginine derivative which was the best had the same internalization ability as the octaarginine. The Dabcyl group increased the internalization of the hexa- and octaarginine bicyclic peptides. The Dabcyl-Cys-(Arg)_4_-Cys-(Arg)_4_-Cys-Lys(Cf) TBMB peptide showed the best internalization compared to the octaarginine. Its internalization was 3-fold better than the octaarginine. (Figure 6)

### 3.3. Investigation of Endocytic Pathways of Entry

**Figure 7:**
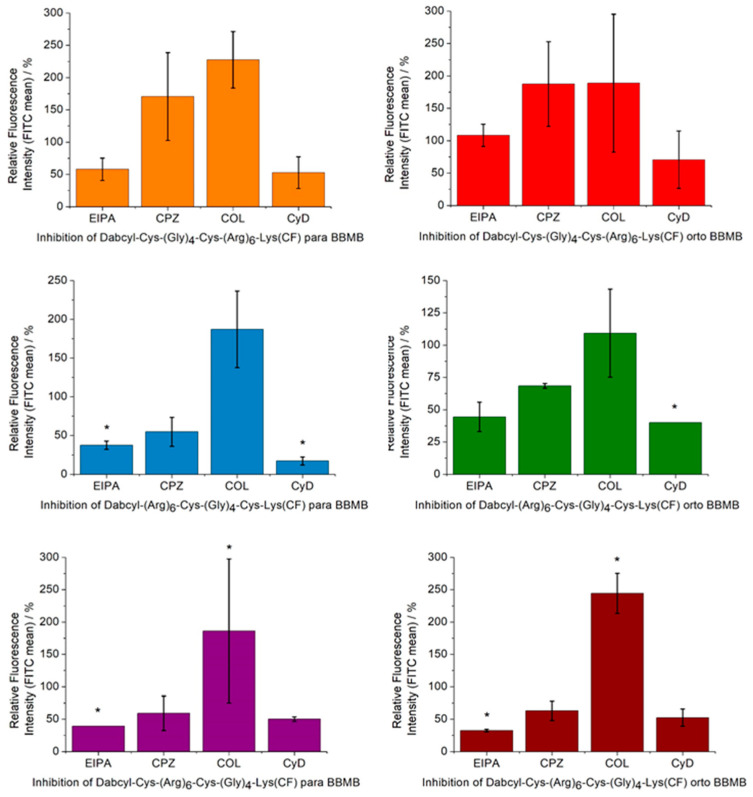
Effect of endocytosis inhibitors on the cellular uptake of peptides. Before the treatment with the peptide conjugates (5 μM) for 90 min, the EBC-1 cells were pretreated with inhibitors EIPA (50 μM), CPZ (30 μM), CyD (5 mM), and COL (10 mM) for 30 min. Significant difference from the control was measured using Student’s *t*-test (* *p* < 0.05). Data represent the mean ± standard deviation (SD).

The CPPs can use several pathways to enter the cell. The dependence of the cellular uptake was measured for each Dabcyl-containing cyclic BBMB peptide using different inhibitors (Figure 7). The 5-(N-ethyl-N-isopropyl)amiloride (EIPA) inhibits macropinocytosis [34], chlorpromazine (CPZ) inhibits clathrin-mediated endocytosis [35], and colchicine (COL) was used to inhibit microtubule formation [36]. Finally, methyl-beta-cyclodextrin (CyD) was used to inhibit caveolae-mediated endocytosis [37].

The treatment with EIPA and CyD significantly reduced the uptake of the peptides. There is one exception, the Dabcyl-Cys-(Gly)_4_-Cys-(Arg)_6_-Lys(Cf) orto BBMB. In that case, the EIPA did not have an effect on the cellular uptake. The CPZ inhibits only the Dabcyl-Cys-(Arg)_6_-Cys-(Gly)_4_-Lys(Cf) orto and para BBMB and Dabcyl-(Arg)_6_-Cys-(Gly)_4_-Cys-Lys(Cf) orto and para BBMB. The treatment with COL did not inhibit cellular uptake.

### 3.4. In Vitro Cytostatic Effect of Conjugates

The peptides can enter the cells remarkably; therefore, we tested the peptides as transport molecules. The two best peptides (Dabcyl-Cys-(Arg)_6_-Cys-(Gly)_4_-Lys orto and para BBMB) were conjugated with DauSuc, an antitumor drug at the ε-amino group of lysine at C-terminus. The Dabcyl-containing DauSuc conjugates showed increased activity on other cell lines [20]. The in vitro cytostatic activity was measured on EBC-1 cells (Table 2).

In terms of DauSuc delivery, the peptides were able to inhibit the proliferation of cancer cells. The Dabcyl-Cys-(Arg)_6_-Cys-(Gly)_4_-Lys(DauSuc) orto BBMB was better but not significantly with IC_50_ value 17.8 µM.

### 3.5. Stability Measurement of Linear and Cyclic Peptides

The analysis was carried out based on the details described in Yuchen Hu et al. [38]. The linear (Dabcyl-Met-(Arg)_6_-Met-(Gly)_4_-Lys(Cf)) and cyclic (Dabcyl-Cys-(Arg)_6_-Cys-(Gly)_4_-Lys(Cf) para BBMB) peptides were incubated with 25% human plasma at 37 °C. An aliquot of the peptide; human plasma mixture was taken at time points 0, 1, and 24 h. The samples were analyzed by RP-HPLC. The two peptides were stable in these conditions (Supplementary Materials, Figures S53–S54); however, the linear peptide started to degrade after 24 h.

## 4. Discussion

The cyclization that enhances rigidity and stability was already shown to increase the internalization of different peptides [30,33]. The distance between the guanidino groups in linear peptides had a strong effect on the internalization [39], but the same effect can be reached by cyclization without the incorporation of other amino acids [40]. In this work, the effect of different cyclization—disulfide bond formation and with different rigid aromatic scaffolds—was examined in combination with the Dabcyl group on the internalization of short oligoarginine-containing peptides. The influence of the position of oligoarginines on internalization was also investigated.

In the case of a cycle with a disulfide bond, the cyclization causes rigidity and thus may enhance cellular uptake. If benzene scaffolds are used to synthesize cyclic or bicyclic peptides, not only the rigidity but the aromatic ring may also enhance the internalization. A linear peptide with methionines instead of cysteines was used to mimic the thioether bond in the cyclic peptide. Our initial findings showed that the cyclization through disulfide bridge (Acyl-Cys-(Arg)_6_-Cys-(Gly)_4_-Lys(Cf)) did increase the internalization of acylated linear peptide Acyl-Met-(Arg)_6_-Met-(Gly)_4_-Lys(Cf) (Figure 2 and Figure 3). When the Dabcyl group was attached to the linear peptide (Dabcyl-Met-(Arg)_6_-Met-(Gly)_4_-Lys(Cf)), it increased the cellular uptake dramatically; 8–10 fold higher internalization than octaarginine (Figure 2). It is in well-accordance with our earlier findings that the Dabcyl group enhances the internalization of hexaarginine, a weak cell-penetrating peptide [20]. Our results prove that this hexarginine derivative is able to deliver short peptide (Gly_4_) into the cells. When the Dabcyl group was attached to the cyclic peptide (Dabcyl-Cys((Arg)_6_-Cys)-(Gly)_4_-Lys(Cf)), enhanced internalization was detected too, but it was less pronounced than in the case of a linear peptide (Figure 3). It seems that the cyclization decreased the efficiency; however, it is still better than the Cf-Arg_8_. These results suggest that both modifications increase the internalization; Dabcyl is better in this aspect, while they do not have a synergistic effect.

Benzene scaffolds (di- and tribromomethylene benzene) were used to synthesize cyclic or bicyclic peptides forming thioether bonds between the thiol groups in the peptide and the benzene ring. For the cyclization, the peptides contain two cysteines, and the position of the hexaarginine and tetraglycine was altered. The cellular uptake was not dependent on the size of the ring; the orto and para derivatives had the same efficiency (Figure 4). The arrangement of the carrier and cargo peptides did not have an effect on it either. Based on the cellular uptake, it turned out that the disulfide bond-formed cycle resulted in a more efficient cyclic peptide. Although in our earlier studies, the aromatic moiety, indole ring in the side chain [24], or benzene ring in the backbone [25,26] could increase the internalization, in this form, the benzene ring is used as a scaffold, and does not have an increasing effect on the internalization.

The attachment of the Dabcyl group to these cyclic peptides with aromatic scaffold enhanced dramatically their cellular uptake (15–25 fold increase) (Figure 5). All peptides were more efficient than the octaarginine (3–6 fold higher uptake). Two peptides (Dabcyl-Cys-(Arg)_6_-Cys-(Gly)_4_-Lys(Cf) para BBMB and Dabcyl-Cys-(Arg)_6_-Cys-(Gly)_4_-Lys(Cf) orto BBMB) had outstanding internalization ability at all concentrations, and they were very active at low concentrations (1 and 5 µM). In these constructs, the hexaarginine was in the cycle. If its position was changed, there was a small decrease in the cellular uptake independently of that at the N- and C-terminus of the structure.

The comparison between the Dabcyl group modified cyclic peptides and the Dabcyl-Met-(Arg)_6_-Met-(Gly)_4_-Lys(Cf) can show that the cyclic peptides have less cellular uptake at 5 and 10 µM concentrations. It seems that there are some structural requirements of the Dabcyl group that caused an enhancing effect, which does not fit in the constrained cyclic form. However, all of these derivatives are better than the octaarginine. So, although some efficiency is lost because of cyclization, its advantage, the increased stability, may compensate for this.

In the case of bicyclic peptides, the Dabcyl-Cys-(Arg)_4_-Cys-(Arg)_4_-Cys-Lys(Cf) TBMB has better internalization compared to the octaarginine while the acylated version (Acyl-Cys-(Arg)_4_-Cys-(Arg)_4_-Cys-Lys(Cf) TBMB) has similar internalization. The tetra- and hexaarginine derivatives have the same but lower internalization (Figure 6). These results showed that if the arginines are in a separated cycle, their effect could not sum up; the bicyclic peptide with six arginines did not show higher internalization than the tetraarginine derivative. However, they have the same internalization as the cyclic peptide with an aromatic scaffold. As the Dabcyl group did not increase the internalization of bicycle peptides with four or six arginine units, it is also supposed that the Dabcyl group has some structural requirements for the internalization enhancement.

The pathway of the internalization of arginine-rich peptides was extensively studied. In some studies, clathrin-mediated endocytosis seemed to be the primary pathway [41,42]; others have reported that it is macropinocytosis [43,44]. In our earlier work, hexaarginne with the Dabcyl group was shown to internalize by micropinocytosis on HeLa cells [20] or caveolae/lipid raft-mediated endocytosis on this cell line [26]. The internalization mechanism of our cyclic peptides with aromatic scaffolds and hexaarginine units was affected by inhibitors (Figure 7). The most effective derivatives in which the hexaarginine is in the cycle showed macropinocytosis and caveolae/lipid raft-mediated endocytosis dependence. It means that in addition to the Dabcyl-hexaarginine used caveolae/lipid raft-mediated endocytosis pathway [26], the cyclization and/or the aromatic ring resulted in a new pathway. Our earlier tetraarginine derivatives modified by aromatic groups showed similar caveolae/lipid raft-mediated endocytosis [24,25,26]. The usage of caveolae/lipid raft-mediated endocytosis could be very positive for the peptides because this route transfers the vesicles and their cargo to the Golgi apparatus and endoplasmic reticulum (ER) instead of the lysosomes [45]. In this way, the peptides can avoid becoming entrapped and degraded by the lysosome and can remain intact, allowing them to transport different cargo, such as drug molecules and proteins. It is interesting that if the hexaarginine was not in the cycle, the picture was changed. In the case of the N-terminal position (the ring was formed in the C-terminal part), all pretreatment inhibited the internalization, while in the case of the C-terminal position, the effect was different on the internalization of para and orto conjugates. The cellular uptake of para derivative depended on the macropinocytosis and caveolae/lipid raft-mediated endocytosis, but any inhibitors did not result in lower uptake in the case of a para derivative. These observations may reflect that although the efficacy of the internalization does not depend on the arrangement of the components, the pathway of cellular uptake of cell-penetrating peptide conjugates could.

A preliminary experiment was done to compare the stability of linear and cyclic peptides. Both derivatives were stable in 25% human plasma, but degradation products appeared in the case of the linear peptide after 24 h treatment. These results suggest that not only the cyclization may enhance the stability but the N-terminal modifications as well [46].

The best two cyclic peptides were conjugated with DauSuc (Dabcyl-Cys-(Arg)_6_-Cys-(Gly)_4_-Lys(DauSuc) orto BBMB and Dabcyl-Cys-(Arg)_6_-Cys-(Gly)_4_-Lys(DauSuc) para BBMB). Both conjugates have antitumor activity, which proves that they are able to transport efficiently impermeable small molecules (DauSuc) into the cell. These results suggest that these peptide conjugates are good candidates for further study. Our plan is to do in vivo experiment in the future with the best constructs.

## 5. Conclusions

In this work, cyclic short cell-penetrating peptides were synthesized. The cyclization was done by disulfide bond formation, which is a commonly used strategy to prepare cyclic peptides, or by using rigid aromatic scaffolds. These scaffolds have the advantage of changing the hydrophobicity of peptides and giving new possible interacting surfaces with the cell membrane. In addition to cyclization, the effect of N-terminal modification by the Dabcyl group was studied, too. These peptides exceeded the uptake by far of octaarginine, the well-known CPP. In bicyclic peptides, the octaarginine-containing peptide was 3-fold better than the standard. While in the case of cyclic peptides with BBMB, all of the peptides were better than the octaarginine. In this case, the best peptides were the ones in which the hexaarginine is in the cycle; their internalization was 5-fold better than the used standard. Additionally, these peptides use favorable uptake routes. While the Dabcyl group-caused enhancing effect was decreased to a small extent by cyclization, the increased enzymatic stability of these constructs can balance this. In this work, our modifications produced effective and more stable CPPs that can be considered for further applications.

## Figures and Tables

**Table 1:** Chemical characterization of peptide conjugates.

Sequence	R_t_	M_calc_	M_meas_
Cf-Arg_8_	11.6	1624.14	1623.86
Acyl-Met-(Arg)_6_-Met-(Gly)_4_-Lys(Cf)	12.8	1971.95	1971.97
Dabcyl-Met-(Arg)_6_-Met-(Gly)_4_-Lys(Cf)	15.0	2181.05	2181.10
Acyl-Cys((Arg)_6_-Cys)-(Gly)_4_-Lys(Cf)	11.9	1913.88	1913.77
Dabcyl-Cys((Arg)_6_-Cys)-(Gly)_4_-Lys(Cf)	13.8	2122.97	2123.01
Dabcyl-Cys-(Arg)_2_-Cys-(Arg)_2_-Cys-Lys(Cf) TBMB	15.9	1801.75	1801.70
Dabcyl-Cys-(Arg)_3_-Cys-(Arg)_3_-Cys-Lys(Cf) TBMB	14.4	2113.95	2113.85
Dabcyl-Cys-(Arg)_4_-Cys-(Arg)_4_-Cys-Lys(Cf) TBMB	13.9	2426.16	2425.55
Acyl-Cys-(Arg)_2_-Cys-(Arg)_2_-Cys-Lys(Cf) TBMB	12.5	1592.66	1592.28
Acyl-Cys-(Arg)_3_-Cys-(Arg)_3_-Cys-Lys(Cf) TBMB	12.0	1904.86	1904.44
Acyl-Cys-(Arg)_4_-Cys-(Arg)_4_-Cys-Lys(Cf) TBMB	11.4	2217.06	2217.07
Dabcyl-Cys-(Gly)_4_-Cys-(Arg)_6_-Lys(Cf) para BBMB	14.7	2227.03	2227.10
Dabcyl-Cys-(Gly)_4_-Cys-(Arg)_6_-Lys(Cf) orto BBMB	14.8	2227.03	2227.07
Dabcyl-Cys-(Arg)_6_-Cys-(Gly)_4_-Lys(Cf) para BBMB	14.2	2227.03	2227.01
Dabcyl-Cys-(Arg)_6_-Cys-(Gly)_4_-Lys(Cf) orto BBMB	14.1	2227.03	2226.99
Dabcyl-(Arg)_6_-Cys-(Gly)_4_-Cys-Lys(Cf) para BBMB	13.6	2227.03	2226.94
Dabcyl-(Arg)_6_-Cys-(Gly)_4_-Cys-Lys(Cf) orto BBMB	13.5	2227.03	2227.05
Acyl-Cys-(Gly)_4_-Cys-(Arg)_6_-Lys(Cf) para BBMB	12.2	2017.94	2017.94
Acyl-Cys-(Gly)_4_-Cys-(Arg)_6_-Lys(Cf) orto BBMB	12.4	2017.94	2017.90
Acyl-Cys-(Arg)_6_-Cys-(Gly)_4_-Lys(Cf) para BBMB	12.4	2017.94	2017.88
Acyl-Cys-(Arg)_6_-Cys-(Gly)_4_-Lys(Cf) orto BBMB	12.2	2017.94	2017.94
Acyl-(Arg)_6_-Cys-(Gly)_4_-Cys-Lys(Cf) para BBMB	12.4	2017.94	2017.86
Acyl-(Arg)_6_-Cys-(Gly)_4_-Cys-Lys(Cf) orto BBMB	12.6	2017.94	2017.95
Dabcyl-Cys-(Arg)_6_-Cys-(Gly)_4_-Lys(DauSuc) para BBMB	16.4	2477.97	2478.08
Dabcyl-Cys-(Arg)_6_-Cys-(Gly)_4_-Lys(DauSuc) orto BBMB	16.5	2477.97	2478.13

The analytical chromatogram was recorded using Hypersil Hypurity C18 column (4.6 mm × 150 mm, 5 μm, 190 Å) with linear gradient elution (0 min 0% B, 2 min 0% B, 22 min 90% B) at 1 mL/min flow rate. The absorbance was measured at λ = 220 nm. The Bruker Amazon SL (Germany) mass spectrometer was used to perform the mass spectrometric analysis. The samples were dissolved in acetonitrile–water (50:50, *v*/*v*) containing 0.1% formic acid.

**Table 2:** Cytostatic activity of peptide/drug conjugates on EBC-1.

Conjugate	IC50 ± SD (µM) *
	EBC-1
Dabcyl-Cys-(Arg)_6_-Cys-(Gly)_4_-Lys(DauSuc) orto BBMB	17.8 ± 2.2
Dabcyl-Cys-(Arg)_6_-Cys-(Gly)_4_-Lys(DauSuc) para BBMB	19.8 ± 0.1

* The cells were incubated with the compound for 3 h, and subsequently cultured in a serum-containing medium for three days. The IC_50_ values were determined by Alamar-blue assay as described in the text. Standard deviation values (SD) are also presented.

## Data Availability

Not applicable.

## Supplementary Materials

The following supporting information can be downloaded at: https://www.mdpi.com/article/10.3390/pharmaceutics15061736/s1, Figures S1–S25: HPLC chromatogram of compounds. Figures S26–S50: MS Spectrum of compounds. Figures S51 and S52: Determination of the IC_50_ values of peptide conjugates by Alamar-blue (Resazurim) assay. Figures S52 and S53: Stability measurement in 25% human plasma.
