# Supplementary material for: Enhancing Cell Penetration Efficiency of Cyclic Oligoarginines Using Rigid Scaffolds
Source: Pharmaceutics. 2023 Jun 14;15(6):1736. doi: 10.3390/pharmaceutics15061736 (PMC10302231; doi:10.3390/pharmaceutics15061736)
Supplement: Supplementary file 1 [file pharmaceutics-15-01736-s001.zip › pharmaceutics-2402021-supplementary.pdf]

pharmaceutics

Supplementary Materials: Enhancing cell penetration efficiency of cyclic oligoarginines using rigid scaffolds

Csaba Bató [1], Ildikó Szabó [2] and Zoltán Bánóczi [1,*]

[1] Department of Organic Chemistry, Institute of Chemistry, Eötvös Loránd University, 1117 Budapest, Hungary; bs.csabi@gmail.com
[2] ELKH-ELTE Research Group of Peptide Chemistry, 1117 Budapest, Hungary; ildiko.szabo@ttk.elte.hu
* Correspondence: zoltan.banoczi@ttk.elte.hu

1. Materials

All amino acid derivatives, Fmoc protected amino acids, N,N'-diisopropylcarbodiimide (DIC), and Rink-amide MBHA resin were purchased from IRIS BiotechGmbH (Marktredwitz, Germany). N,N-diisopropylethylamine (DIEA), 1.8-diazabicyclo[5.4.0]undec-7-ene (DBU), thioanisole were FLUKA (Buchs, Switzerland). Solvents for synthesis and purification were obtained from Molar Chemicals Ltd (Budapest, Hungary). 1,2-bis(bromomethyl)benzene, 1,4-bis(bromomethyl)benzene, 1,3,5-tris(bromomethyl)benzene, Ethyl cyano(hydroxyimino)acetate (Oxima Pure), phenol, 5(6)-carboxyfluorescein (Cf), trifluoroacetic acid (TFA), colchicine (COL), Resazurim (Alamar-blue) and all other chemicals used in biological experiments were purchased from Sigma Aldrich (Hungary). While tris(hydroxymethyl)aminomethane (TRIS) was purchased from VWR (Hungary). Dabcyl was purchased from AAT Bioquest. Chlorpromazine (CPZ), methyl-beta-cyclodextrin (CyD) were purchased from TCI chemicals.

1.1. Synthesis of succinylated daunomycin (DauSuc)

Inclusion of the succinyl linker into daunomycin was performed in a method reported earlier. [1] 100 mg daunomycin×HCl was reacted with 2 eq succinic anhydride in the presence of 3 eq DIEA, the reactants were allowed to mix overnight in 5 ml DMF. The solvent was subsequently evaporated, and the remaining crude product was dissolved in mobile phase B (0.1%TFA in 80% ACN:20% H2O) and further purified with RP-HPLC on a C18 Column.

1.2. RP-HPLC

Analytical RP-HPLC was performed on Exformma (Exformma Technology (ASIA) Co., Ltd, Hong Kong, China) HPLC system. The used column was Hypersil Hypurity C18 column (4.6 mm × 150 mm, 5 µm, 190 Å). Linear gradient elution (0 min 0% B; 2 min 0% B; 22 min 90% B) was used with eluent A (0.1% TFA in water) and eluent B (0.1% TFA in acetonitrile-water (80:20, v/v)) at 1mL/min flowrate, the peaks were detected at λ= 220 nm for both analytical and preparative RP-HPLC. The samples were dissolved in a minimum amount of eluent B and injected into the analytical RP-HPLC. The crude products were purified on a semi-preparative Phenomenex Jupiter C18 column (250 × 10 mm I.D.) with 10 mm silica (300 Å pore size) (Torrance, CA, USA). Flow rate was 4 mL/min and linear gradient elution was applied. The samples were dissolved in eluent A containing small percent eluent B (10–25% depending on sequence).

1 Z. Bánóczi, B. Peregi, E. Orbán, R. Szabó, F. Hudecz, Synthesis of daunomycin-oligoarginine onjugates and their effect on human leukemia cells (HL-60), Arkivoc. 2008 (2008) 140–153.

1.3. Mass spectrometry

The molecular weight of peptides and conjugates was determined with ESI-MS using Bruker Amazon SL (Germany). The samples were dissolved in water-acetonitrile solution (50:50) with 0.1% formic acid. The samples were directly injected with a syringe pump. Parameters: capillary voltage: 4 kV, nebulizer gas: 10 psi, dry gas: 4 L/min, heated capillary temperature: 250 °C.

1.4. Cell Culture

EBC-1 human lung squamous cancer cells (CRL-5889TM) were used for the in vitro analysis. These cells were a generous gift of Prof. László Kőhidai from Semmelweis University, Faculty of Medicine, Department of Genetics, Cell- and Immunobiology. Cells were maintained in Dulbecco's Modified Eagle Medium (DMEM) supplemented with 10% heat inactivated foetal calf serum (FCS), nonessential amino acids (NEAA), sodium pyruvate (1 mM), L-Glutamine (2 mM), 1% nonessential amino acids and 1% penicillin-streptomycin (from 10,000 units penicillin and 10mg/ml streptomycin). Cells were maintained in plastic tissue culture dishes at 37 °C with a humidified atmosphere containing 5% CO_2/95% air.

1.5. Plasma

Healthy volunteer blood specimens were purchased from Hungarian National Blood Transfusion Service (Budapest, Hungary). Blood was collected in BD vacutainer tubes containing sodium citrate as anticoagulant (BD Biosciences, San Jose, CA, USA). The anticoagulated blood was centrifuged at 1200 rpm for 15 min at 4°C. Following the centrifugation, the plasma was transferred into polypropylene tubes and apportioned into 1 mL aliquots. The aliquots were stored at –80°C.

2. Chemical Characterization of Peptide Conjugates

2.1. RP-HPLC

Analytical RP-HPLC was performed on Exformma (Exformma Technology (ASIA) Co., Ltd, Hong Kong, China) HPLC system. The used column was Hypersil Hypurity C18 column (4.6 mm × 150 mm, 5 μm, 190 Å). Linear gradient elution (0 min 0% B; 2 min 0% B; 22 min 90% B) was used with eluent A (0.1% TFA in water) and eluent B (0.1% TFA in acetonitrile-water (80:20, v/v)) at 1mL/min flowrate, the peaks were detected at λ= 220 nm for both analytical and preparative RP-HPLC. The samples were dissolved in a minimum amount of eluent B and injected into the analytical RP-HPLC. The crude products were purified on a semi-preparative Phenomenex Jupiter C18 column (250 × 10 mm I.D.) with 10 mm silica (300 Å pore size) (Torrance, CA, USA). Flow rate was 4 mL/min and linear gradient elution was applied. The samples were dissolved in eluent A containing small percent eluent B (10–25% depending on sequence).

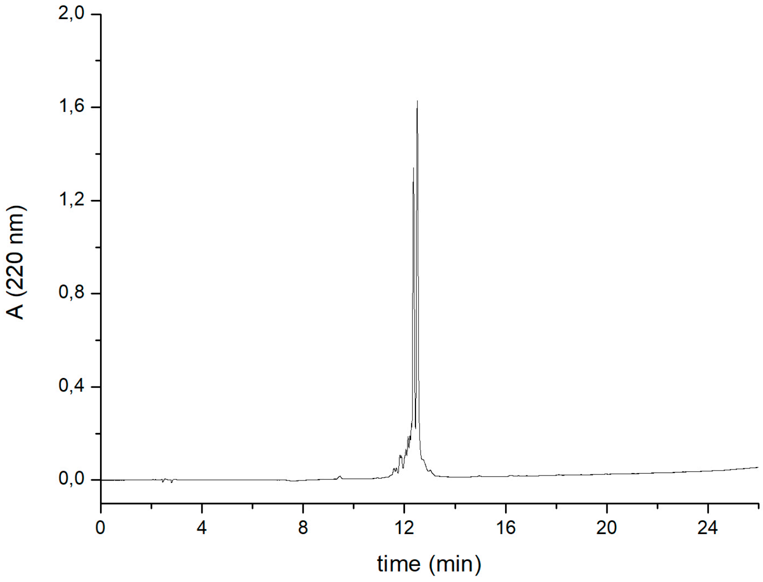

Figure S1. HPLC chromatogram of Acyl-Cys-(Arg)$_2$-Cys-(Arg)$_2$-Cys-Lys(Cf) TBMB. Retention time was obtained on Hypersil Hypurity C18 column (4.6 mm x 150 mm, 5 µm, 190 Å). The applied linear gradient elution was 0 min 0% B, 2 min 0% B, 22 min 90% B at 1 mL/min flow rate. The detection was carried on at λ = 220 nm.

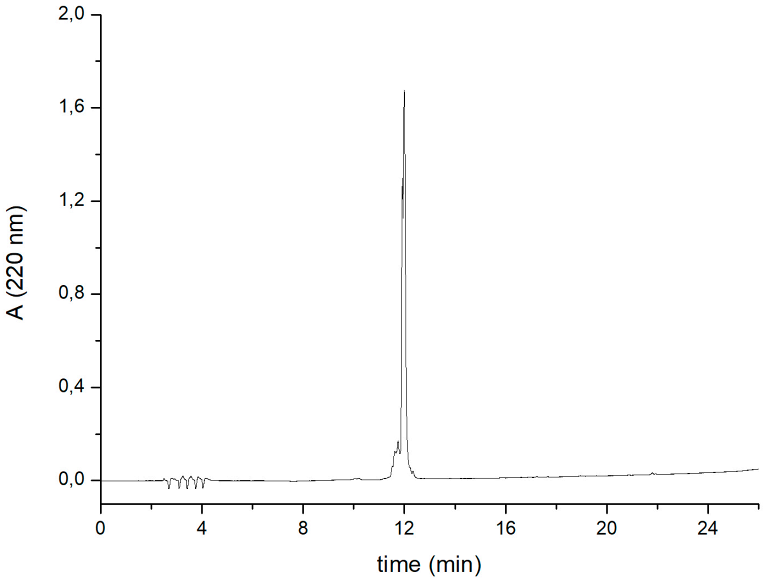

Figure S2. HPLC chromatogram of Acyl-Cys-(Arg)$_3$-Cys-(Arg)$_3$-Cys-Lys(Cf) TBMB. Retention time was obtained on Hypersil Hypurity C18 column (4.6 mm x 150 mm, 5 µm, 190 Å). The applied linear gradient elution was 0 min 0% B, 2 min 0% B, 22 min 90% B at 1 mL/min flow rate. The detection was carried on at λ = 220 nm.

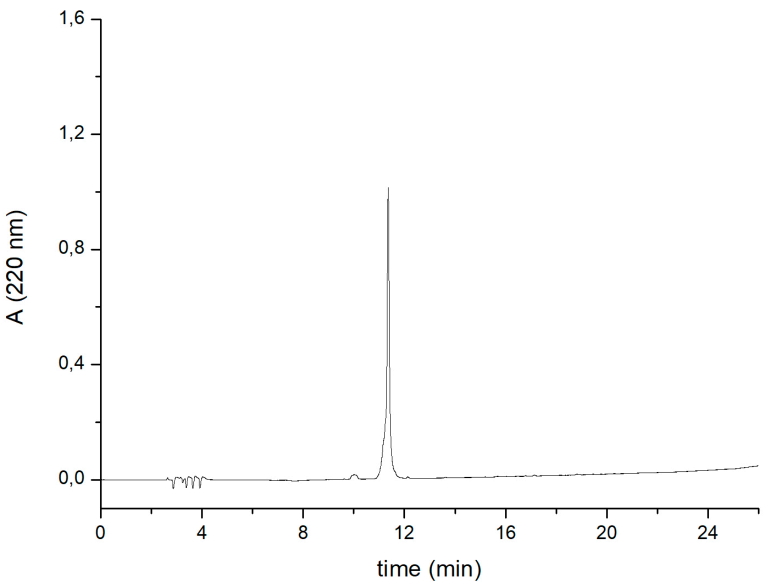

Figure S3. HPLC chromatogram of Acyl-Cys-(Arg)$_4$-Cys-(Arg)$_4$-Cys-Lys(Cf) TBMB. Retention time was obtained on Hypersil Hypurity C18 column (4.6 mm x 150 mm, 5 µm, 190 Å). The applied linear gradient elution was 0 min 0% B, 2 min 0% B, 22 min 90% B at 1 mL/min flow rate. The detection was carried on at λ = 220 nm.

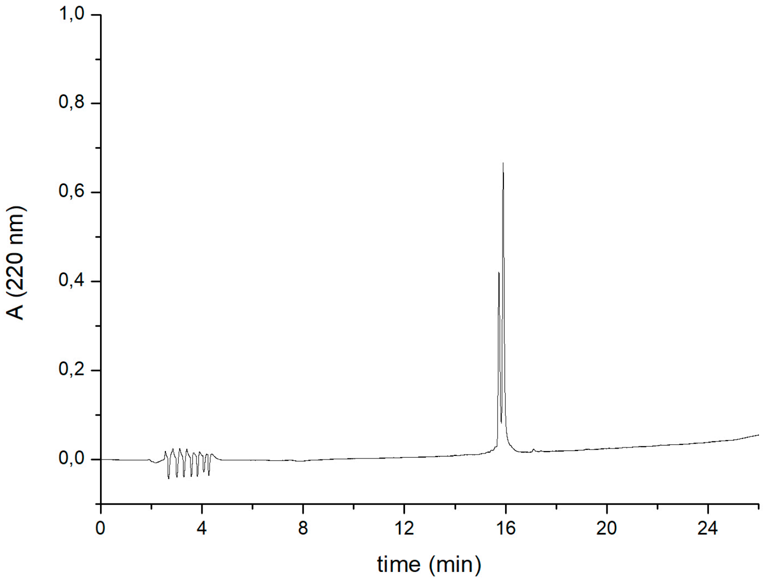

Figure S4. HPLC chromatogram of Dabcyl-Cys-(Arg)$_2$-Cys-(Arg)$_2$-Cys-Lys(Cf) TBMB. Retention time was obtained on Hypersil Hypurity C18 column (4.6 mm x 150 mm, 5 µm, 190 Å). The applied linear gradient elution was 0 min 0% B, 2 min 0% B, 22 min 90% B at 1 mL/min flow rate. The detection was carried on at λ = 220 nm.

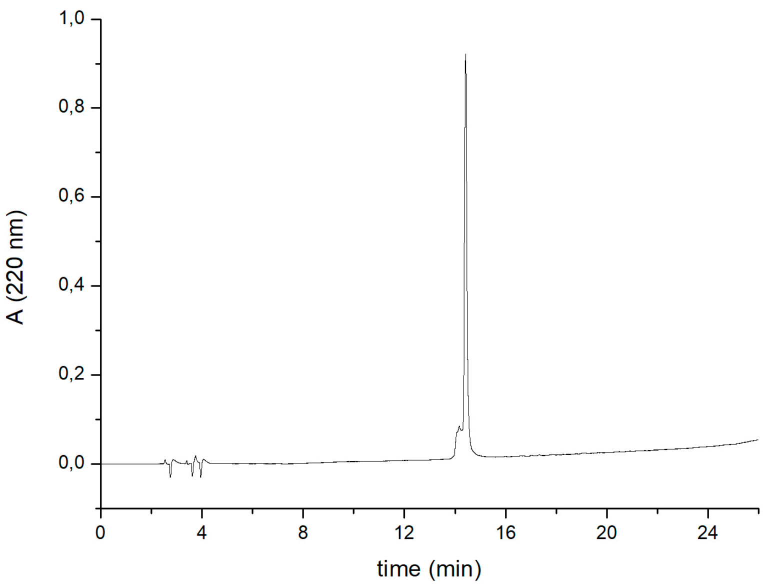

Figure S5. HPLC chromatogram of Dabcyl-Cys-(Arg)3-Cys-(Arg)3-Cys-Lys(Cf) TBMB. Retention time was obtained on Hypersil Hypurity C18 column (4.6 mm x 150 mm, 5 µm, 190 Å). The applied linear gradient elution was 0 min 0% B, 2 min 0% B, 22 min 90% B at 1 mL/min flow rate. The detection was carried on at λ = 220 nm.

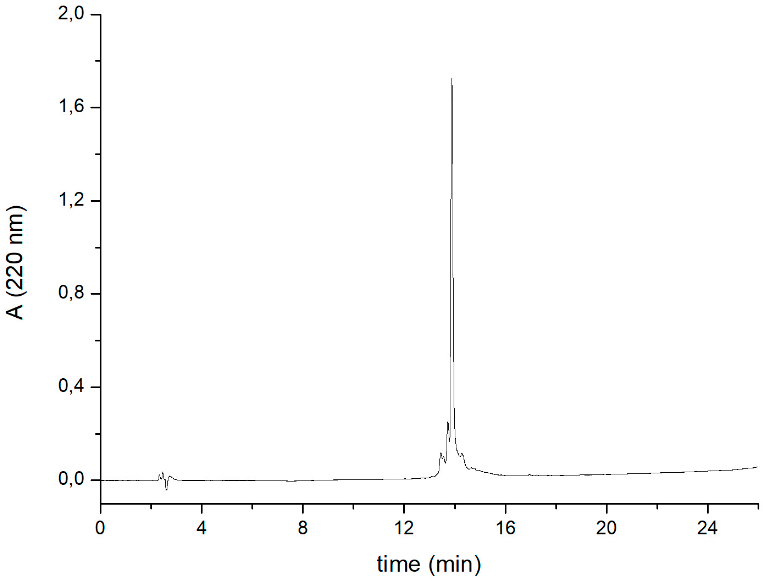

Figure S6. HPLC chromatogram of Dabcyl-Cys-(Arg)4-Cys-(Arg)4-Cys-Lys(Cf) TBMB. Retention time was obtained on Hypersil Hypurity C18 column (4.6 mm x 150 mm, 5 µm, 190 Å). The applied linear gradient elution was 0 min 0% B, 2 min 0% B, 22 min 90% B at 1 mL/min flow rate. The detection was carried on at λ = 220 nm.

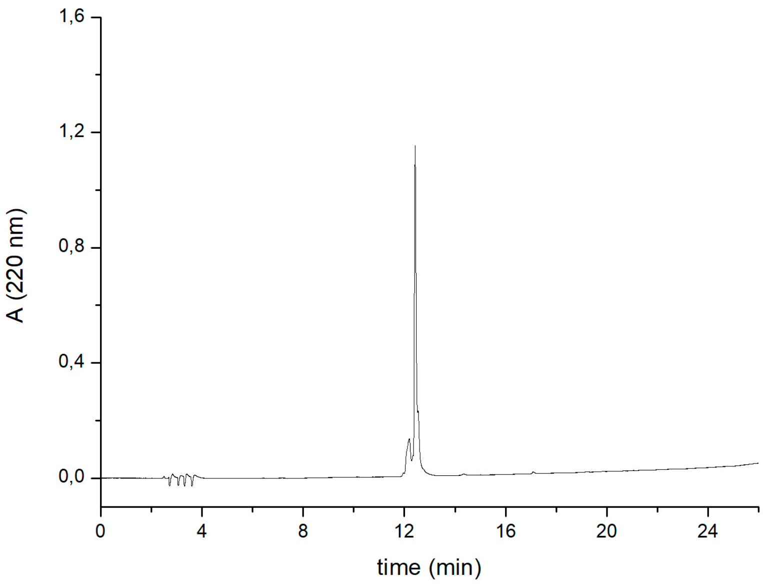

Figure S7. HPLC chromatogram of Acyl-Cys-(Gly)$_4$-Cys-(Arg)$_6$-Lys(Cf) orto BBMB. Retention time was obtained on Hypersil Hypurity C18 column (4.6 mm x 150 mm, 5 μm, 190 Å). The applied linear gradient elution was 0 min 0% B, 2 min 0% B, 22 min 90% B at 1 mL/min flow rate. The detection was carried on at λ = 220 nm.

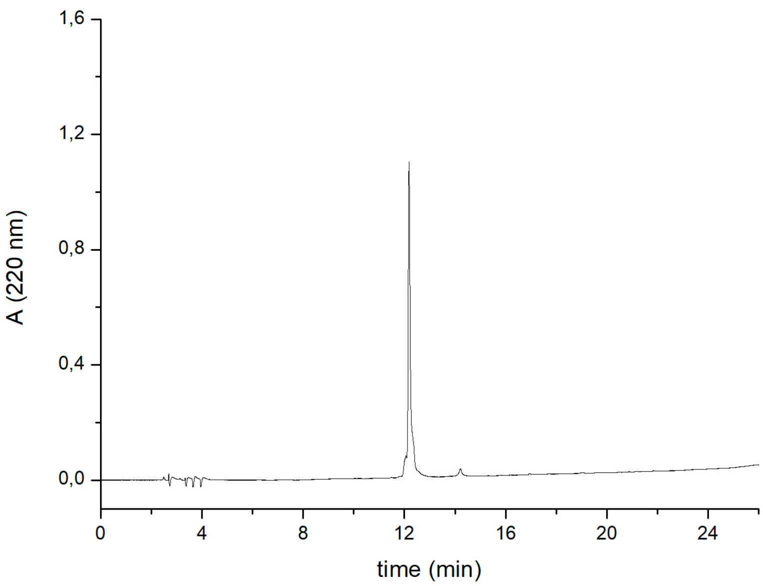

Figure S8. HPLC chromatogram of Acyl-Cys-(Gly)$_4$-Cys-(Arg)$_6$-Lys(Cf) para BBMB. Retention time was obtained on Hypersil Hypurity C18 column (4.6 mm x 150 mm, 5 μm, 190 Å). The applied linear gradient elution was 0 min 0% B, 2 min 0% B, 22 min 90% B at 1 mL/min flow rate. The detection was carried on at λ = 220 nm.

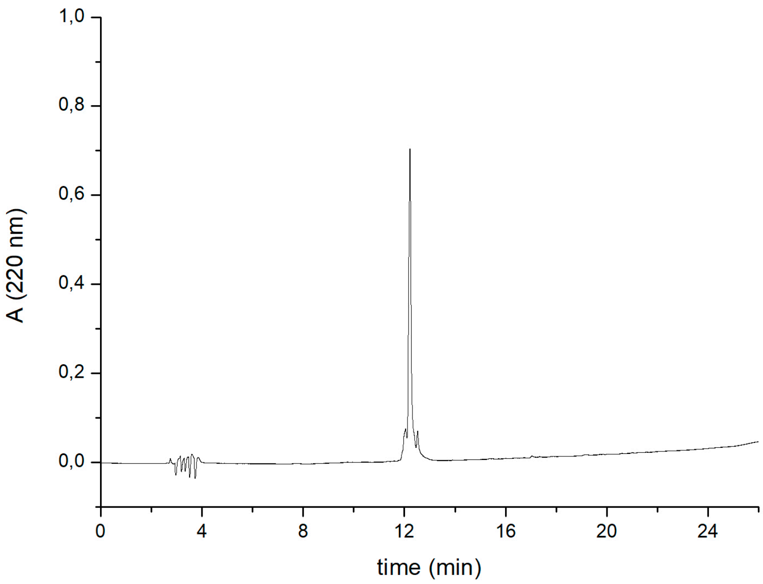

Figure S9. HPLC chromatogram of Acyl-Cys-(Arg)6-Cys-(Gly)4-Lys(Cf) orto BBMB. Retention time was obtained on Hypersil Hypurity C18 column (4.6 mm x 150 mm, 5 µm, 190 Å). The applied linear gradient elution was 0 min 0% B, 2 min 0% B, 22 min 90% B at 1 mL/min flow rate. The detection was carried on at λ = 220 nm.

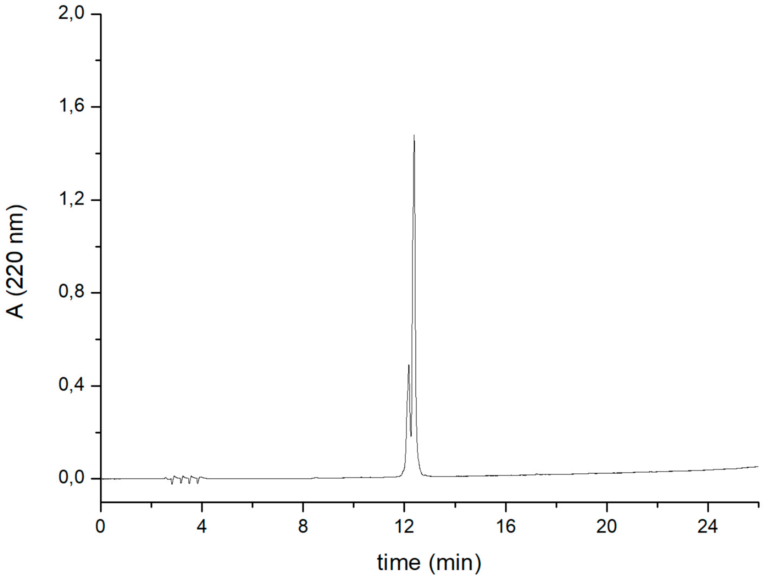

Figure S10. HPLC chromatogram of Acyl-Cys-(Arg)6-Cys-(Gly)4-Lys(Cf) para BBMB. Retention time was obtained on Hypersil Hypurity C18 column (4.6 mm x 150 mm, 5 µm, 190 Å). The applied linear gradient elution was 0 min 0% B, 2 min 0% B, 22 min 90% B at 1 mL/min flow rate. The detection was carried on at λ = 220 nm.

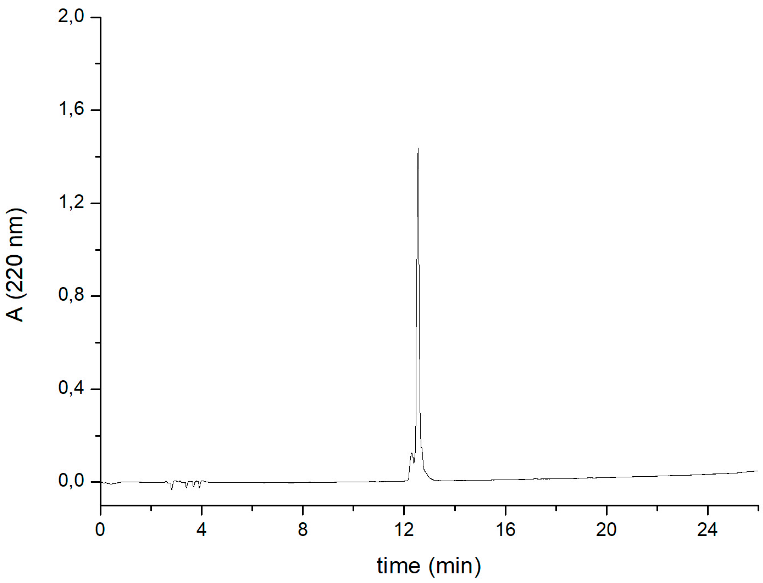

Figure S11. HPLC chromatogram of Acyl-(Arg)$_6$-Cys-(Gly)$_4$-Cys-Lys(Cf) orto BBMB. Retention time was obtained on Hypersil Hypurity C18 column (4.6 mm x 150 mm, 5 µm, 190 Å). The applied linear gradient elution was 0 min 0% B, 2 min 0% B, 22 min 90% B at 1 mL/min flow rate. The detection was carried on at λ = 220 nm.

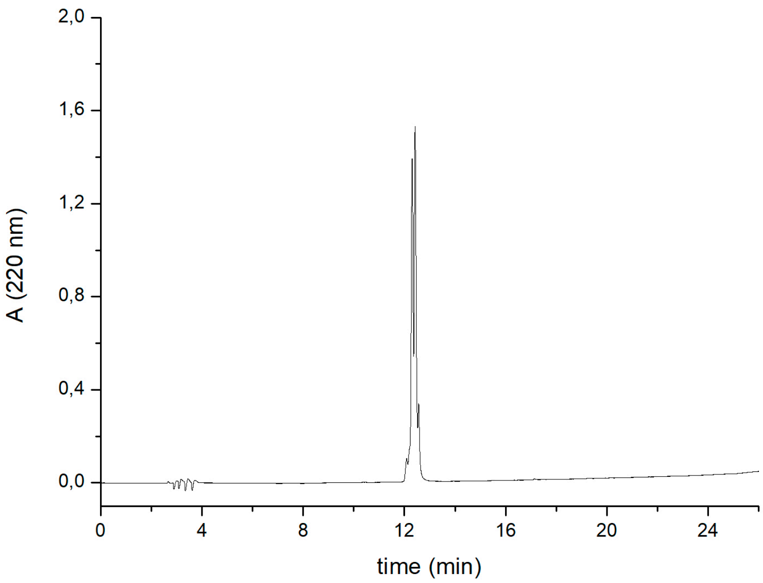

Figure S12. HPLC chromatogram of Acyl-(Arg)$_6$-Cys-(Gly)$_4$-Cys-Lys(Cf) para BBMB. Retention time was obtained on Hypersil Hypurity C18 column (4.6 mm x 150 mm, 5 µm, 190 Å). The applied linear gradient elution was 0 min 0% B, 2 min 0% B, 22 min 90% B at 1 mL/min flow rate. The detection was carried on at λ = 220 nm.

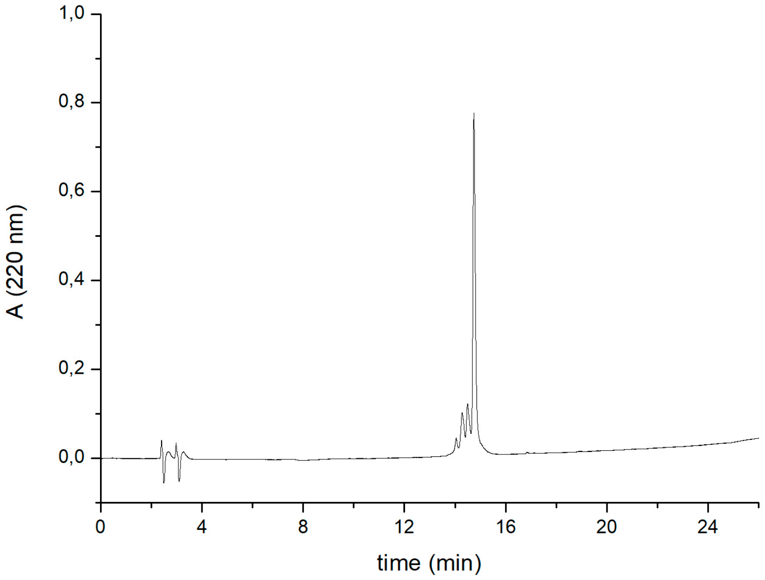

Figure S13. HPLC chromatogram of Dabcyl-Cys-(Gly)$_4$-Cys-(Arg)$_6$-Lys(Cf) orto BBMB. Retention time was obtained on Hypersil Hypurity C18 column (4.6 mm x 150 mm, 5 µm, 190 Å). The applied linear gradient elution was 0 min 0% B, 2 min 0% B, 22 min 90% B at 1 mL/min flow rate. The detection was carried on at λ = 220 nm.

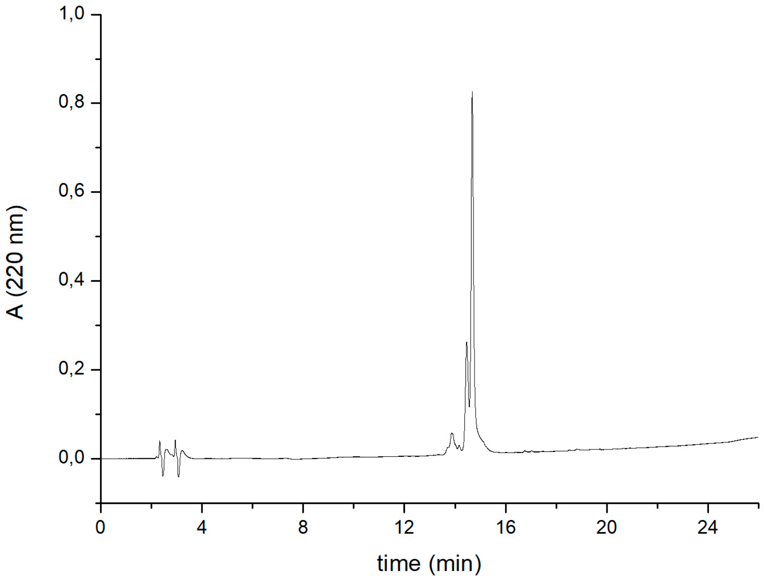

Figure S14. HPLC chromatogram of Dabcyl-Cys-(Gly)$_4$-Cys-(Arg)$_6$-Lys(Cf) para BBMB. Retention time was obtained on Hypersil Hypurity C18 column (4.6 mm x 150 mm, 5 µm, 190 Å). The applied linear gradient elution was 0 min 0% B, 2 min 0% B, 22 min 90% B at 1 mL/min flow rate. The detection was carried on at λ = 220 nm.

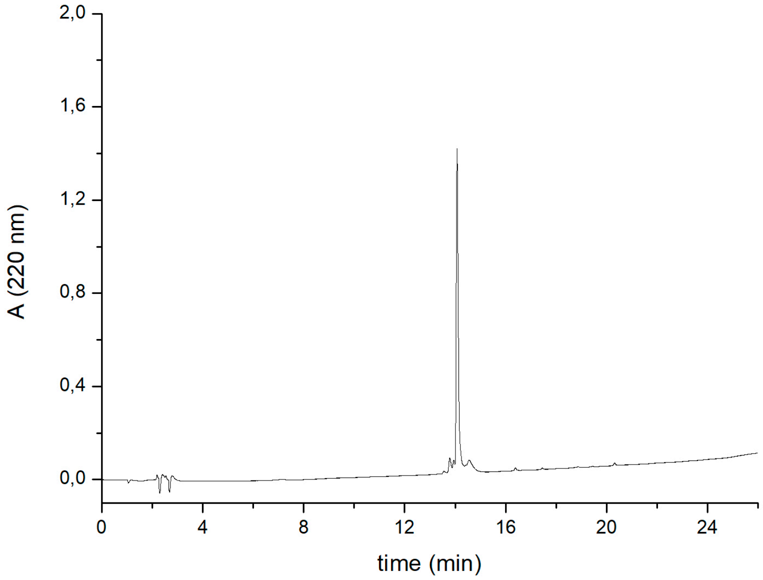

Figure S15. HPLC chromatogram of Dabcyl-Cys-(Arg)$_6$-Cys-(Gly)$_4$-Lys(Cf) orto BBMB. Retention time was obtained on Hypersil Hypurity C18 column (4.6 mm x 150 mm, 5 µm, 190 Å). The applied linear gradient elution was 0 min 0% B, 2 min 0% B, 22 min 90% B at 1 mL/min flow rate. The detection was carried on at λ = 220 nm.

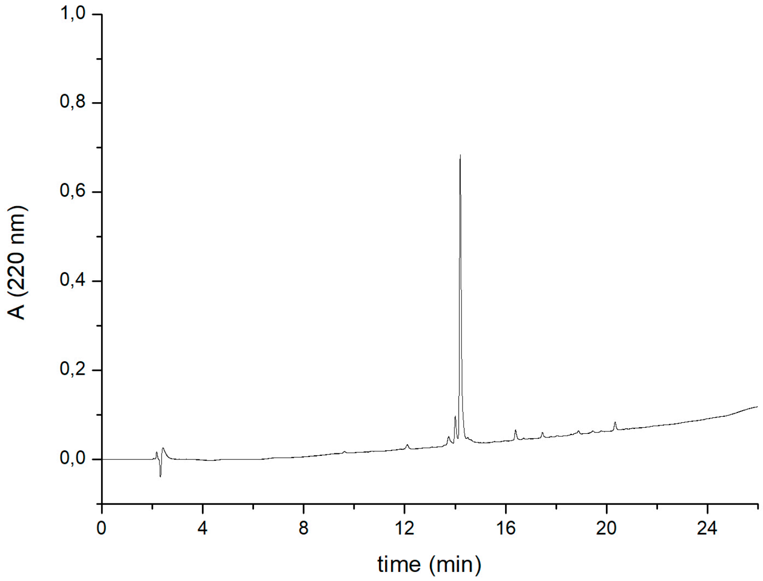

Figure S16. HPLC chromatogram of Dabcyl-Cys-(Arg)$_6$-Cys-(Gly)$_4$-Lys(Cf) para BBMB. Retention time was obtained on Hypersil Hypurity C18 column (4.6 mm x 150 mm, 5 µm, 190 Å). The applied linear gradient elution was 0 min 0% B, 2 min 0% B, 22 min 90% B at 1 mL/min flow rate. The detection was carried on at λ = 220 nm.

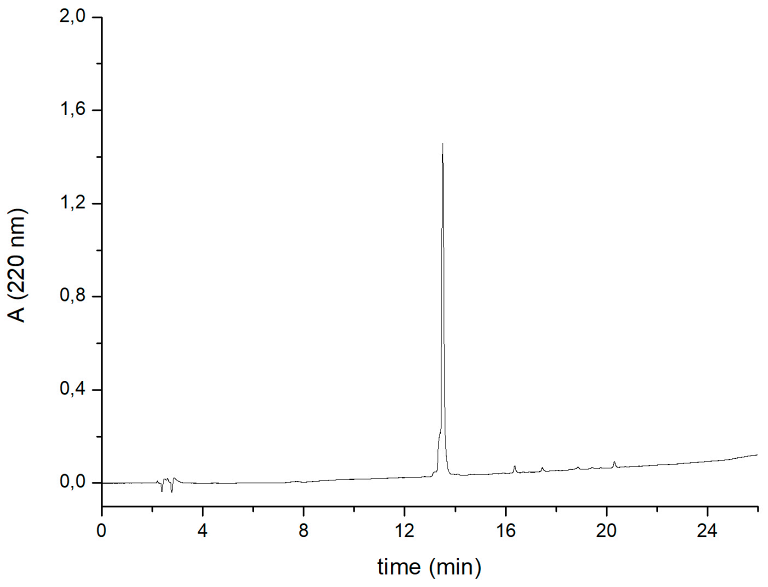

Figure S17. HPLC chromatogram of Dabcyl-(Arg)$_6$-Cys-(Gly)$_4$-Cys-Lys(Cf) orto BBMB. Retention time was obtained on Hypersil Hypurity C18 column (4.6 mm x 150 mm, 5 µm, 190 Å). The applied linear gradient elution was 0 min 0% B, 2 min 0% B, 22 min 90% B at 1 mL/min flow rate. The detection was carried on at λ = 220 nm.

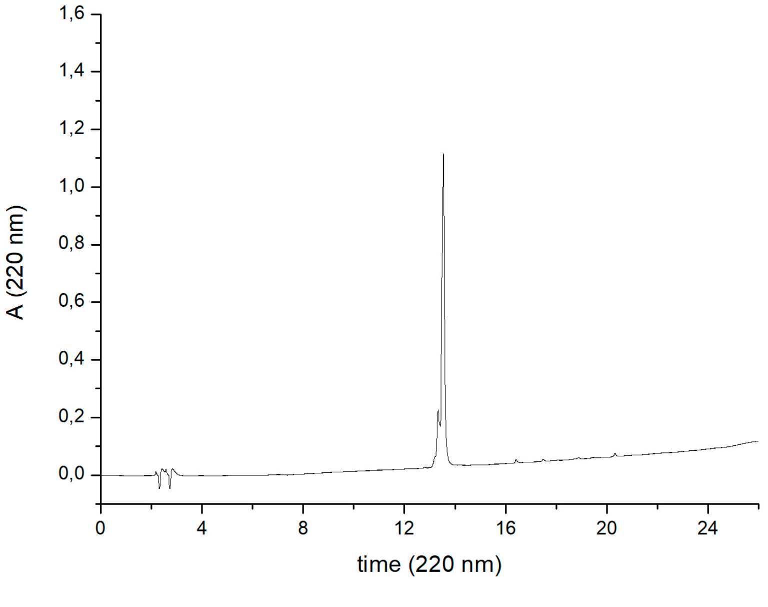

Figure S18. HPLC chromatogram of Dabcyl-(Arg)$_6$-Cys-(Gly)$_4$-Cys-Lys(Cf) para BBMB. Retention time was obtained on Hypersil Hypurity C18 column (4.6 mm x 150 mm, 5 µm, 190 Å). The applied linear gradient elution was 0 min 0% B, 2 min 0% B, 22 min 90% B at 1 mL/min flow rate. The detection was carried on at λ = 220 nm.

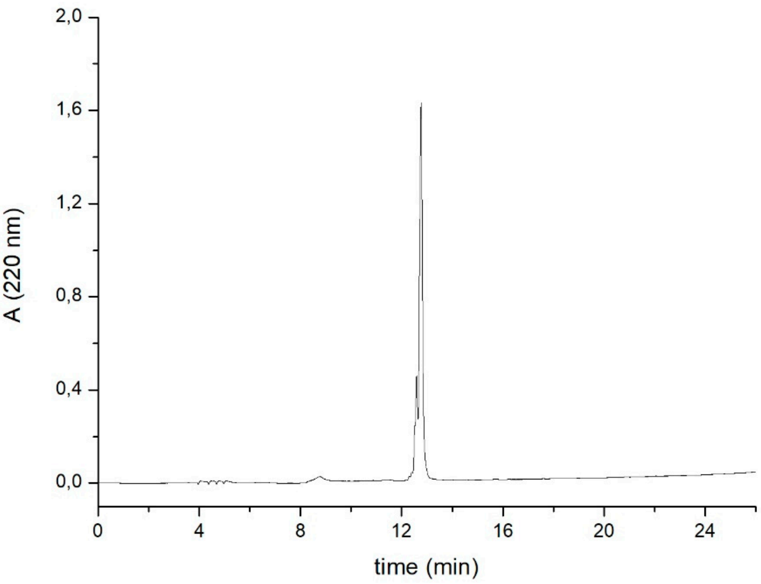

Figure S19. HPLC chromatogram of Acyl-Met-(Arg)$_6$-Met-(Gly)$_4$-Lys(Cf). Retention time was obtained on Hypersil Hypurity C18 column (4.6 mm x 150 mm, 5 µm, 190 Å). The applied linear gradient elution was 0 min 0% B, 2 min 0% B, 22 min 90% B at 1 mL/min flow rate. The detection was carried on at λ = 220 nm.

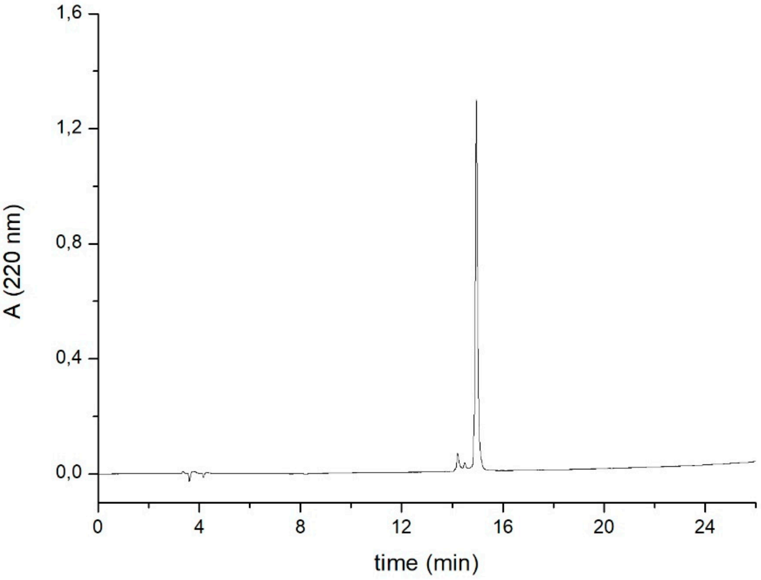

Figure S20. HPLC chromatogram of Dabcyl-Met-(Arg)$_6$-Met-(Gly)$_4$-Lys(Cf). Retention time was obtained on Hypersil Hypurity C18 column (4.6 mm x 150 mm, 5 µm, 190 Å). The applied linear gradient elution was 0 min 0% B, 2 min 0% B, 22 min 90% B at 1 mL/min flow rate. The detection was carried on at λ = 220 nm.

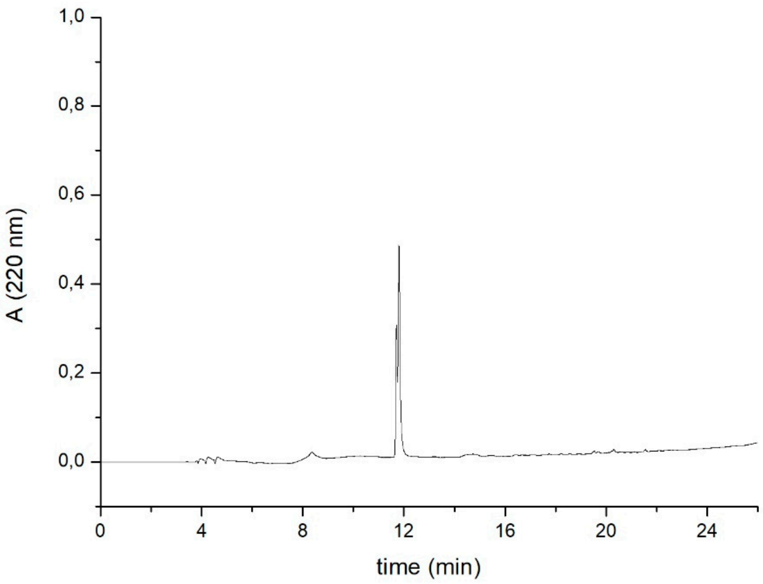

Figure S21. HPLC chromatogram Acyl-Cys((Arg)$_6$-Cys)-(Gly)$_4$-Lys(Cf). Retention time was obtained on Hypersil Hypurity C18 column (4.6 mm x 150 mm, 5 µm, 190 Å). The applied linear gradient elution was 0 min 0% B, 2 min 0% B, 22 min 90% B at 1 mL/min flow rate. The detection was carried on at λ = 220 nm.

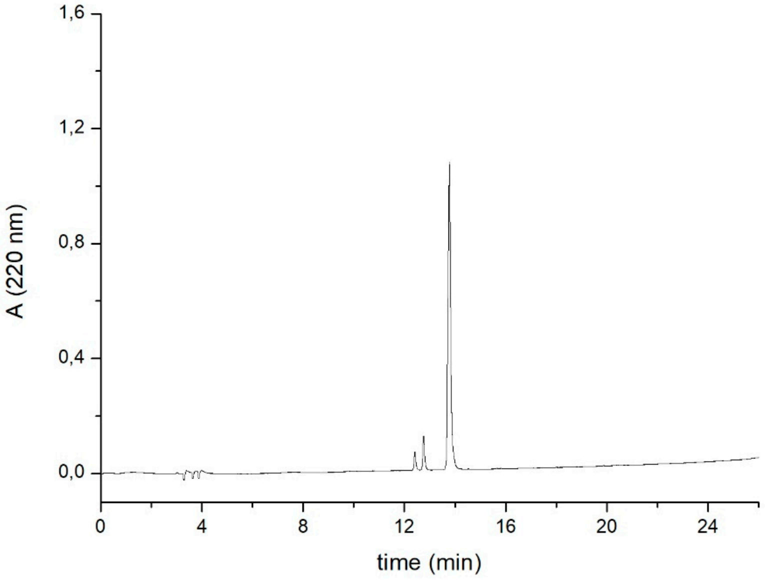

Figure S22. HPLC chromatogram Dabcyl-Cys((Arg)$_6$-Cys)-(Gly)$_4$-Lys(Cf). Retention time was obtained on Hypersil Hypurity C18 column (4.6 mm x 150 mm, 5 µm, 190 Å). The applied linear gradient elution was 0 min 0% B, 2 min 0% B, 22 min 90% B at 1 mL/min flow rate. The detection was carried on at λ = 220 nm.

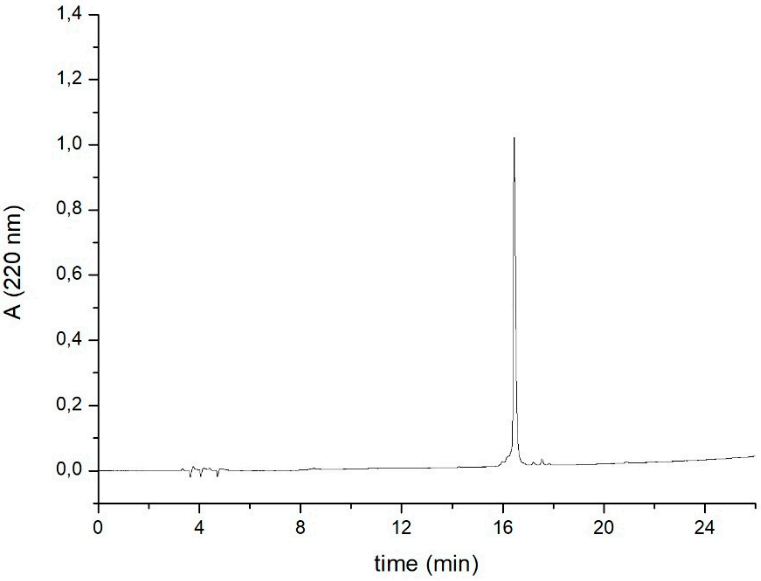

Figure S23. HPLC chromatogram Dabcyl-Cys-(Arg)$_6$-Cys-(Gly)$_4$-Lys(DauSuc) orto BBMB. Retention time was obtained on Hypersil Hypurity C18 column (4.6 mm x 150 mm, 5 μm, 190 Å). The applied linear gradient elution was 0 min 0% B, 2 min 0% B, 22 min 90% B at 1 mL/min flow rate. The detection was carried on at λ = 220 nm.

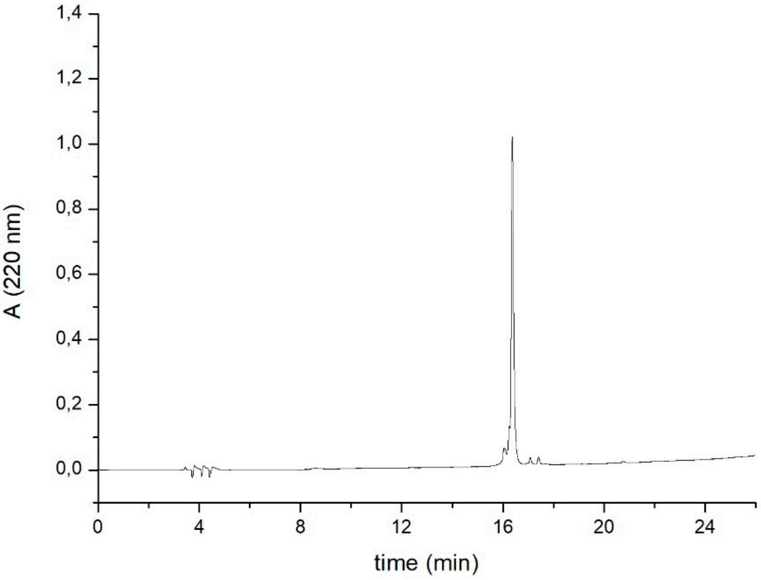

Figure S24. HPLC chromatogram Dabcyl-Cys-(Arg)$_6$-Cys-(Gly)$_4$-Lys(DauSuc) para BBMB. Retention time was obtained on Hypersil Hypurity C18 column (4.6 mm x 150 mm, 5 μm, 190 Å). The applied linear gradient elution was 0 min 0% B, 2 min 0% B, 22 min 90% B at 1 mL/min flow rate. The detection was carried on at λ = 220 nm.

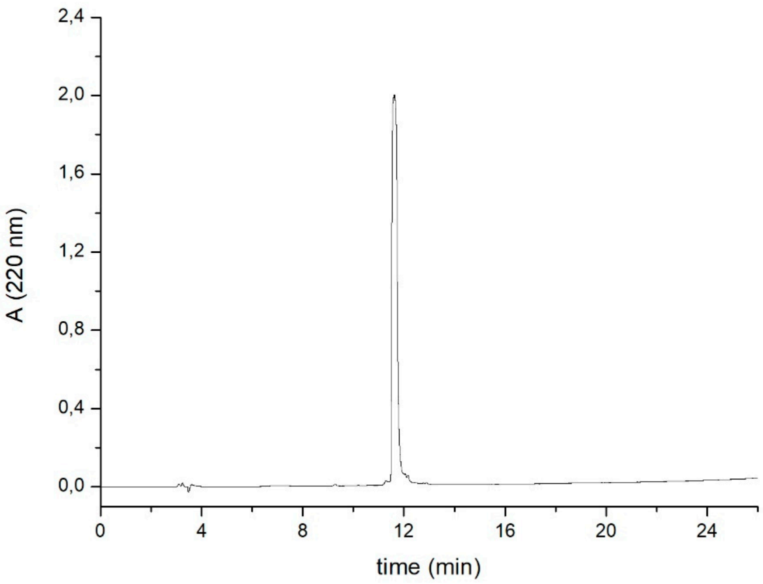

Figure S25. HPLC chromatogram Cf-Arg$_8$. Retention time was obtained on Hypersil Hypurity C18 column (4.6 mm x 150 mm, 5 μm, 190 Å). The applied linear gradient elution was 0 min 0% B, 2 min 0% B, 22 min 90% B at 1 mL/min flow rate. The detection was carried on at λ = 220 nm.

2.2. Mass spectrometry

The molecular weight of peptides and conjugates was determined with ESI-MS using Bruker Amazon SL (Germany). The samples were dissolved in water-acetonitrile solution (50:50) with 0.1% formic acid. The samples were directly injected with a syringe pump. Parameters: capillary voltage: 4 kV, nebulizer gas: 10 psi, dry gas: 4 L/min, heated capillary temperature: 250 °C.

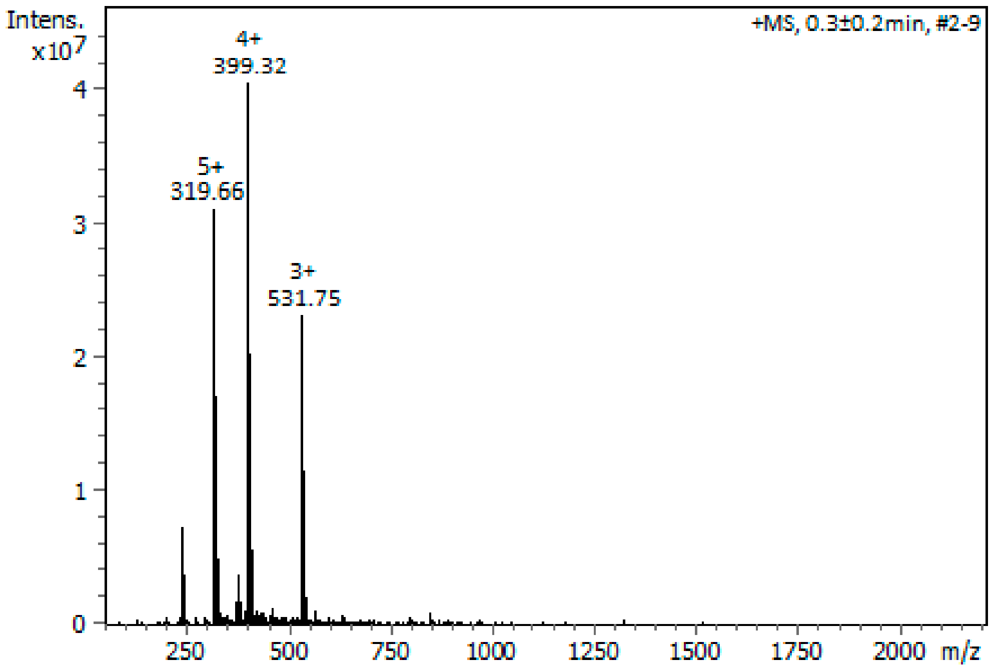

Figure S26. MS Spectrum of Acyl-Cys-$(Arg)_2$-Cys-$(Arg)_2$-Cys-Lys(Cf) TBMB. The identity of the peptide conjugate was determined using Bruker Amazon SL (Germany). The samples are dissolved in water-acetonitrile (50:50) with 0.1% formic acid.

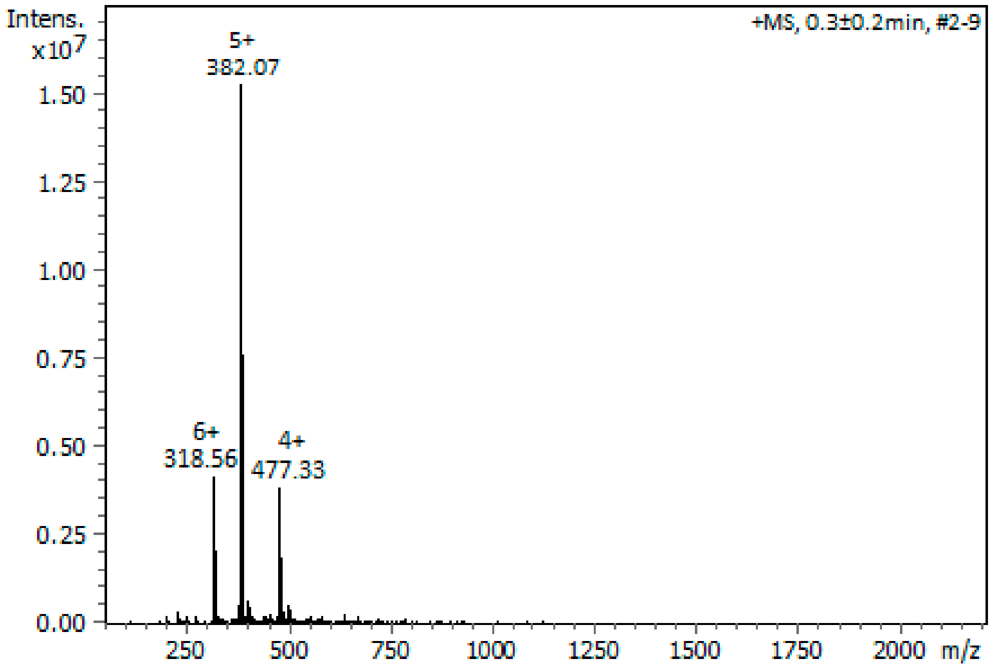

Figure S27. MS Spectrum of Acyl-Cys-$(Arg)_3$-Cys-$(Arg)_3$-Cys-Lys(Cf) TBMB. The identity of the peptide conjugate was determined using Bruker Amazon SL (Germany). The samples are dissolved in water-acetonitrile (50:50) with 0.1% formic acid.

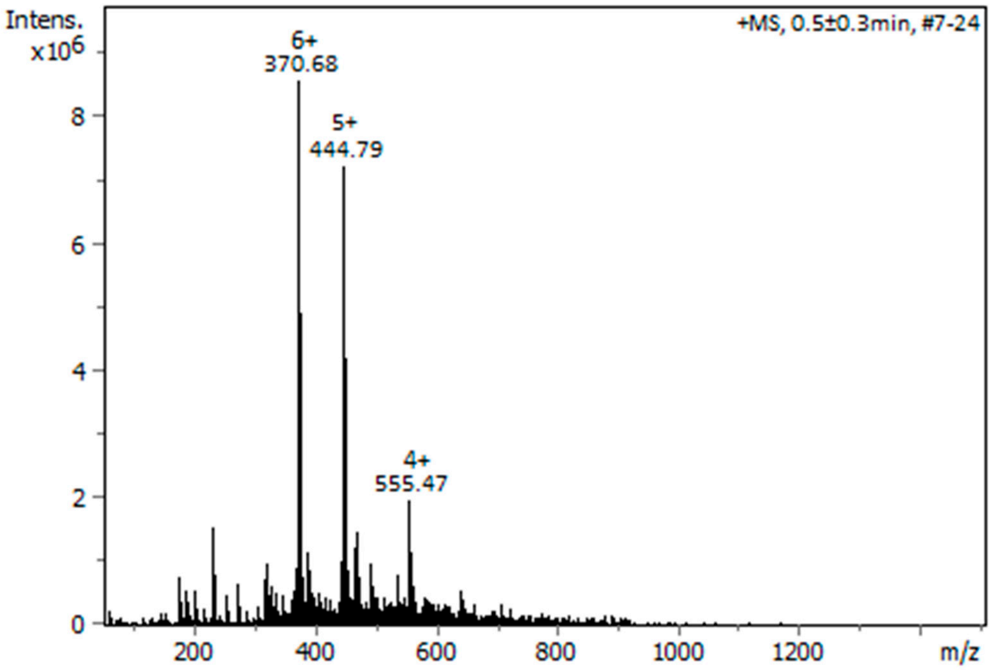

Figure S28. MS Spectrum of Acyl-Cys-(Arg)$_4$-Cys-(Arg)$_4$-Cys-Lys(Cf) TBMB. The identity of the peptide conjugate was determined using Bruker Amazon SL (Germany). The samples are dissolved in water-acetonitrile (50:50) with 0.1% formic acid.

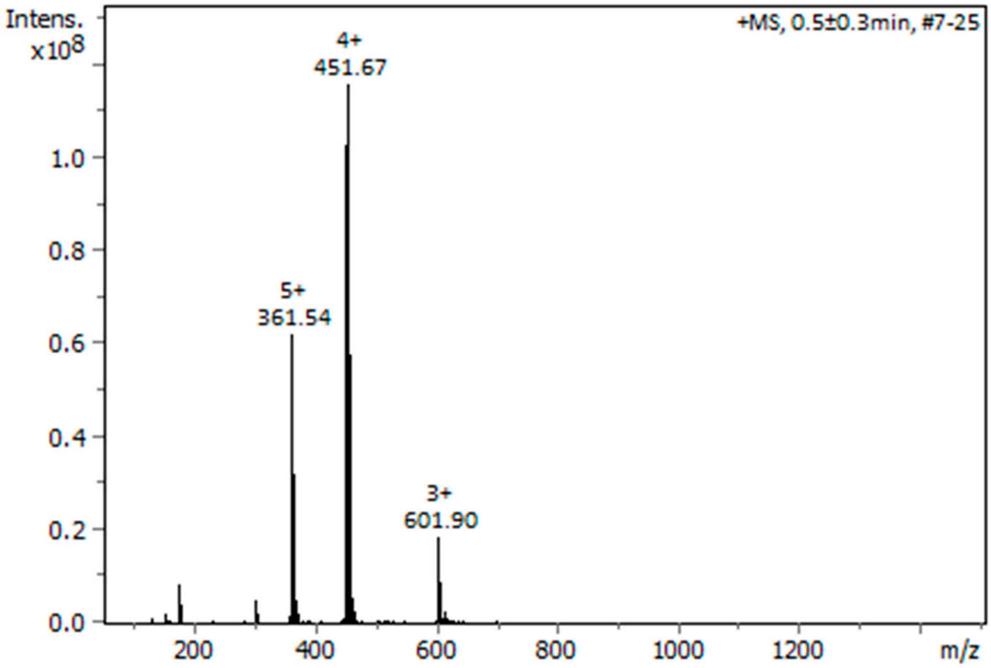

Figure S29. MS Spectrum of Dabcyl-Cys-(Arg)$_2$-Cys-(Arg)$_2$-Cys-Lys(Cf) TBMB. The identity of the peptide conjugate was determined using Bruker Amazon SL (Germany). The samples are dissolved in water-acetonitrile (50:50) with 0.1% formic acid.

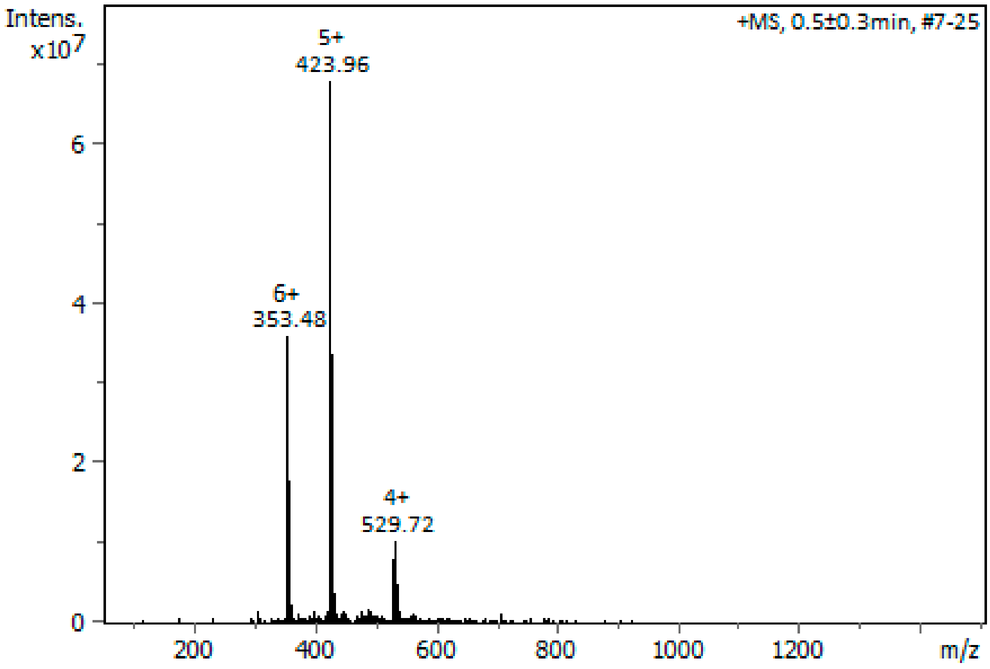

Figure S30. MS Spectrum of Dabcyl-Cys-(Arg)$_3$-Cys-(Arg)$_3$-Cys-Lys(Cf) TBMB. The identity of the peptide conjugate was determined using Bruker Amazon SL (Germany). The samples are dissolved in water-acetonitrile (50:50) with 0.1% formic acid.

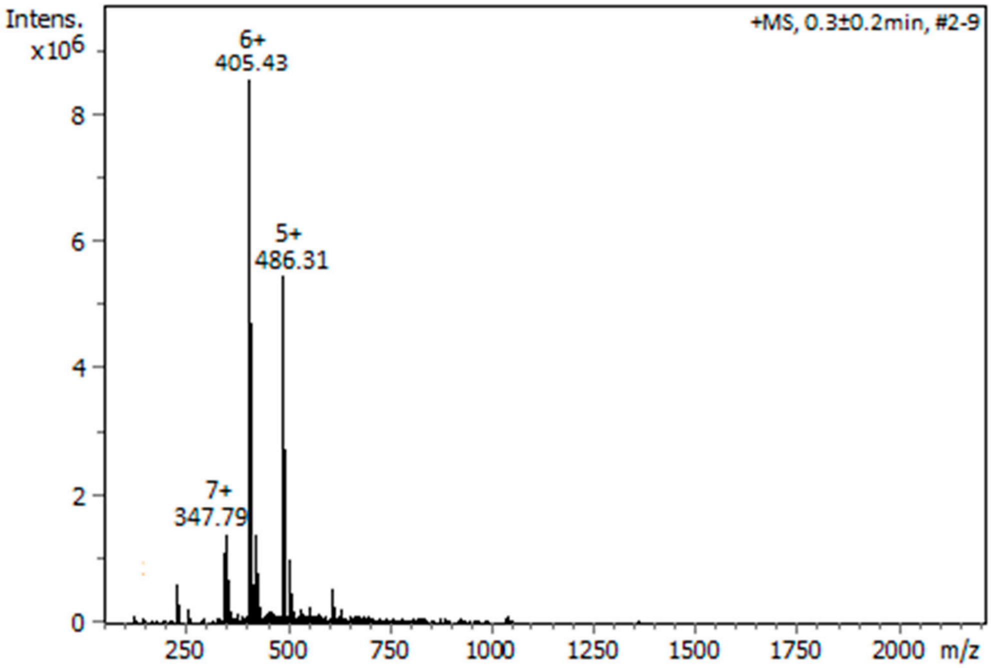

Figure S31. MS Spectrum of Dabcyl-Cys-(Arg)$_4$-Cys-(Arg)$_4$-Cys-Lys(Cf) TBMB. The identity of the peptide conjugate was determined using Bruker Amazon SL (Germany). The samples are dissolved in water-acetonitrile (50:50) with 0.1% formic acid.

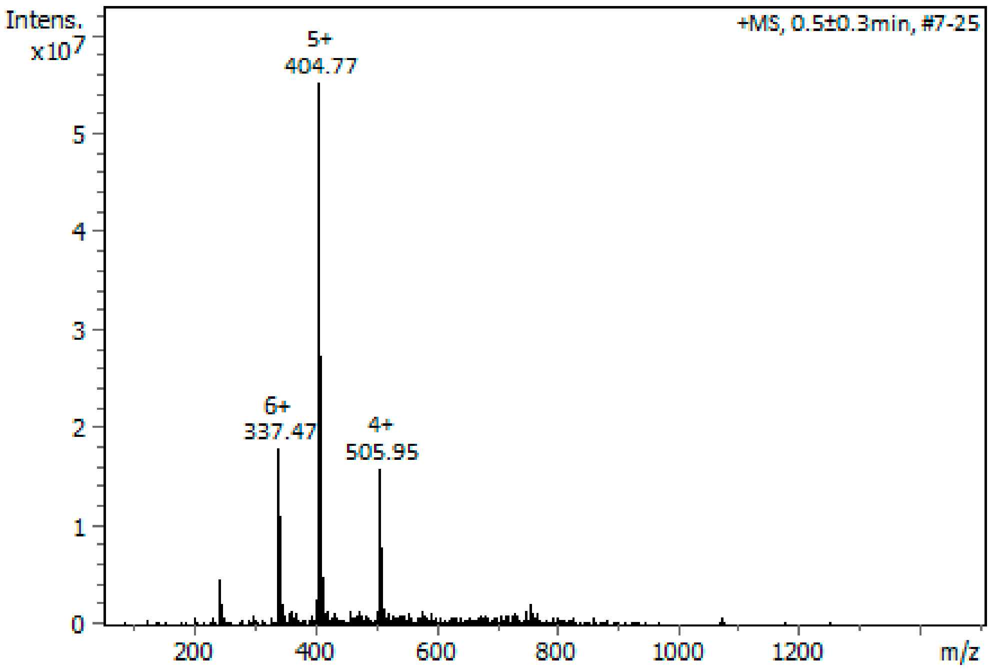

Figure S32. MS Spectrum of Acyl-Cys-(Gly)$_4$-Cys-(Arg)$_6$-Lys(Cf) orto BBMB. The identity of the peptide conjugate was determined using Bruker Amazon SL (Germany). The samples are dissolved in water-acetonitrile (50:50) with 0.1% formic acid.

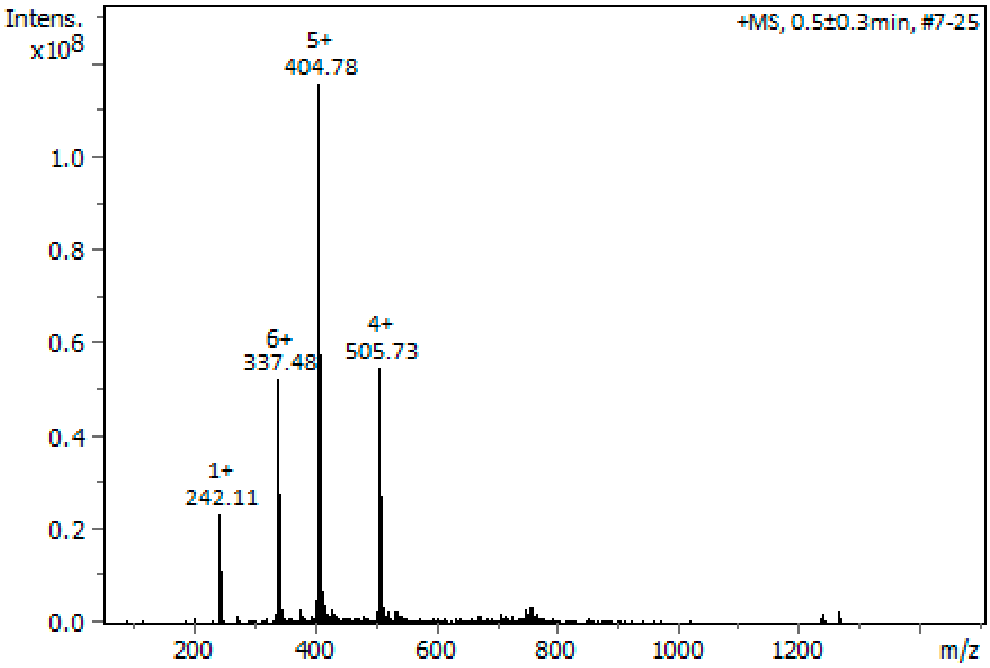

Figure S33. MS Spectrum of Acyl-Cys-(Gly)$_4$-Cys-(Arg)$_6$-Lys(Cf) para BBMB. The identity of the peptide conjugate was determined using Bruker Amazon SL (Germany). The samples are dissolved in water-acetonitrile (50:50) with 0.1% formic acid.

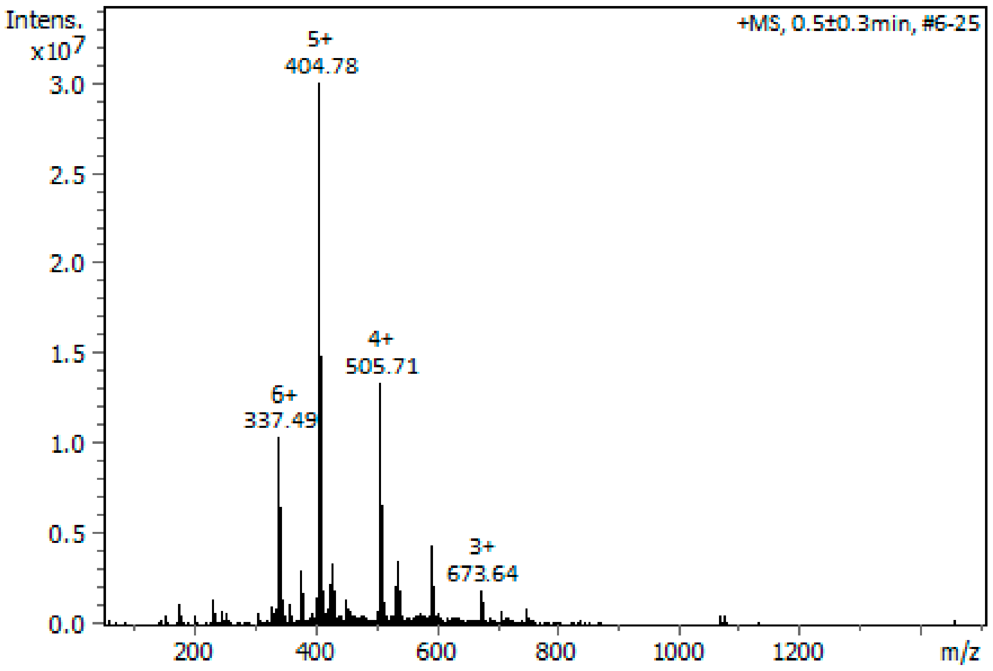

Figure S34. MS Spectrum of Acyl-Cys-(Arg)$_6$-Cys-(Gly)$_4$-Lys(Cf) orto BBMB. The identity of the peptide conjugate was determined using Bruker Amazon SL (Germany). The samples are dissolved in water-acetonitrile (50:50) with 0.1% formic acid.

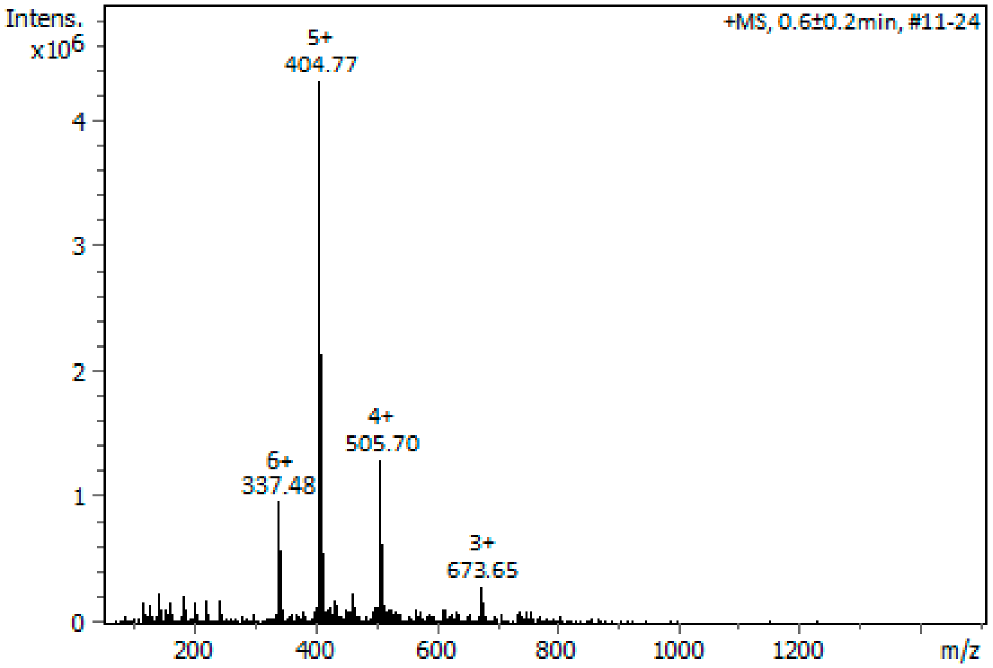

Figure S35. MS Spectrum of Acyl-Cys-(Arg)$_6$-Cys-(Gly)$_4$-Lys(Cf) para BBMB. The identity of the peptide conjugate was determined using Bruker Amazon SL (Germany). The samples are dissolved in water-acetonitrile (50:50) with 0.1% formic acid.

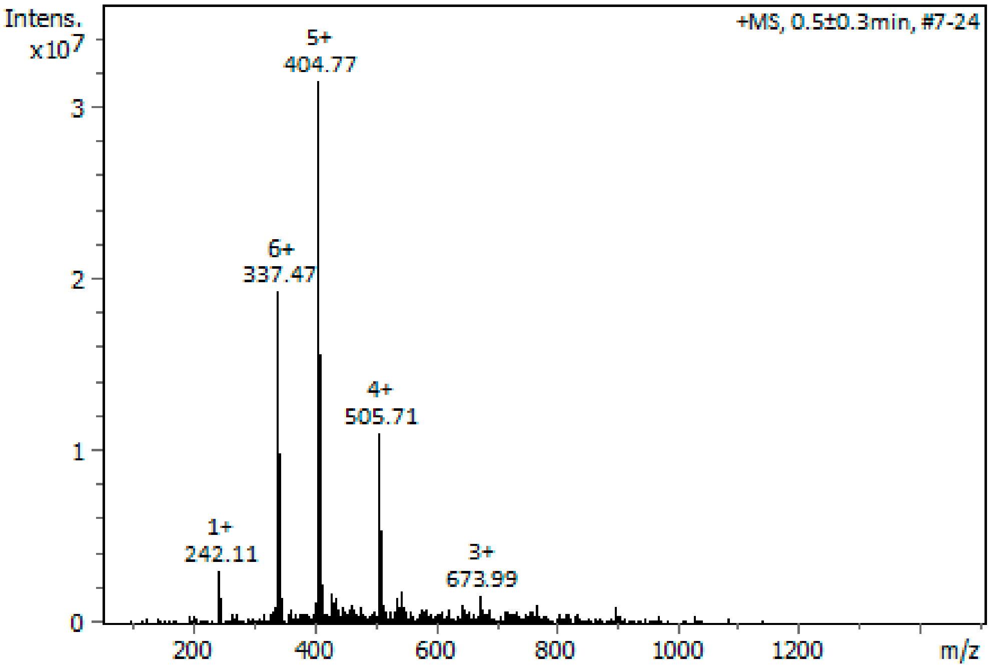

Figure S36. MS Spectrum of Acyl-(Arg)$_6$-Cys-(Gly)$_4$-(Cys)-Lys(Cf) orto BBMB. The identity of the peptide conjugate was determined using Bruker Amazon SL (Germany). The samples are dissolved in water-acetonitrile (50:50) with 0.1% formic acid.

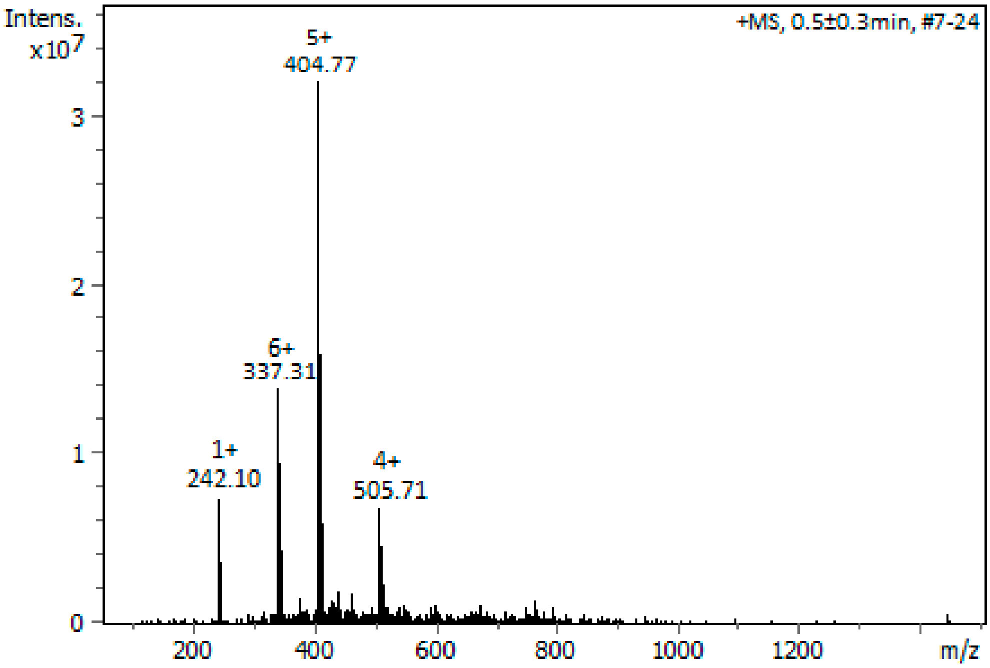

Figure S37. MS Spectrum of Acyl-(Arg)$_6$-Cys-(Gly)$_4$-(Cys)-Lys(Cf) para BBMB. The identity of the peptide conjugate was determined using Bruker Amazon SL (Germany). The samples are dissolved in water-acetonitrile (50:50) with 0.1% formic acid.

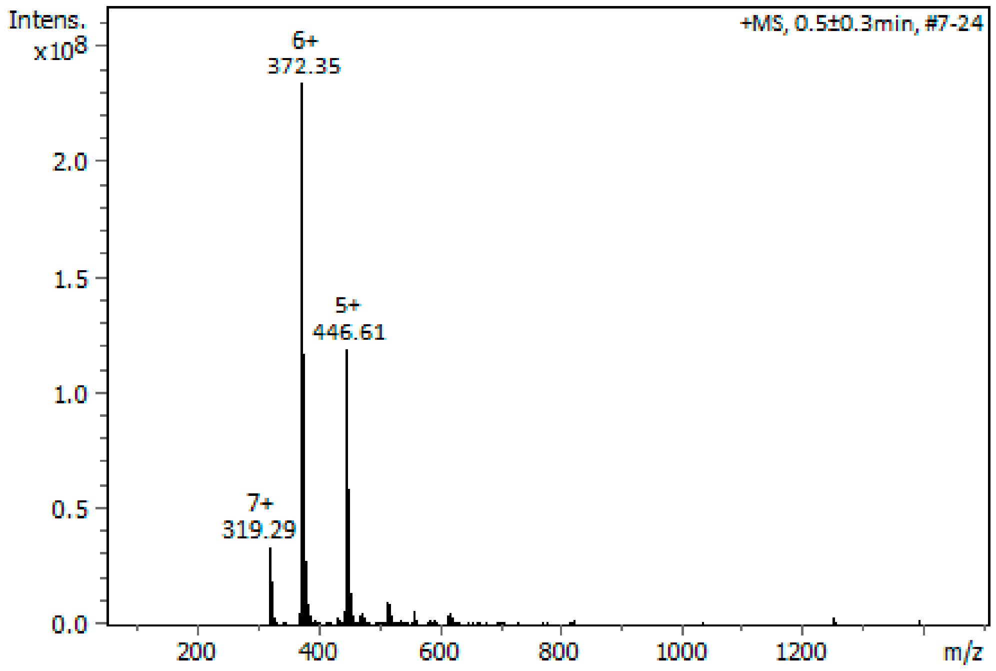

Figure S38. MS Spectrum of Dabcyl-Cys-(Gly)$_4$-Cys-(Arg)$_6$-Lys(Cf) orto BBMB. The identity of the peptide conjugate was determined using Bruker Amazon SL (Germany). The samples are dissolved in water-acetonitrile (50:50) with 0.1% formic acid.

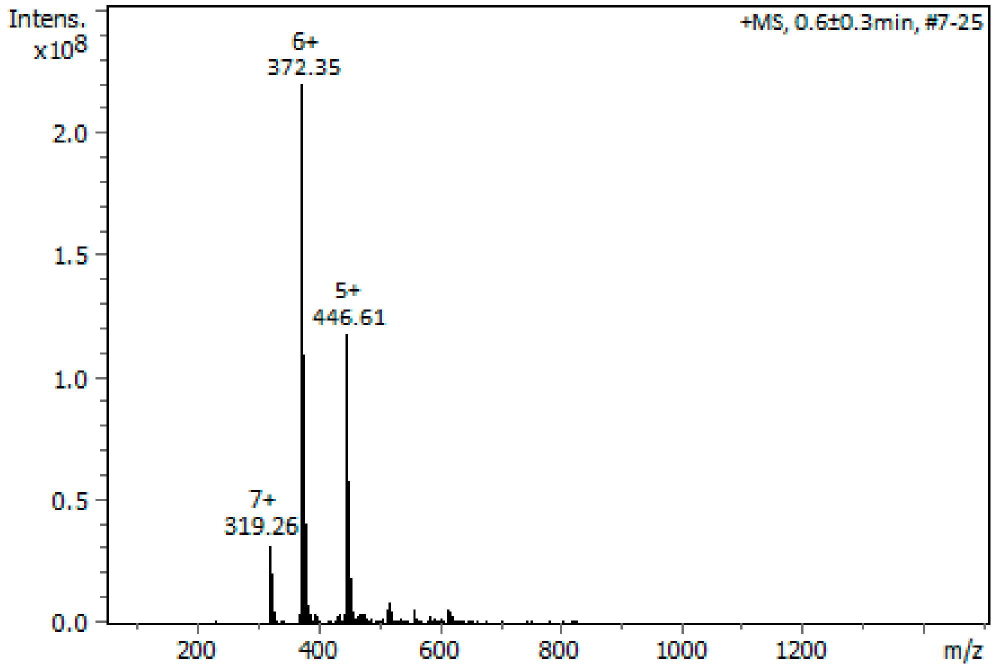

Figure S39. MS Spectrum of Dabcyl-Cys-(Gly)$_4$-Cys-(Arg)$_6$-Lys(Cf) para BBMB. The identity of the peptide conjugate was determined using Bruker Amazon SL (Germany). The samples are dissolved in water-acetonitrile (50:50) with 0.1% formic acid.

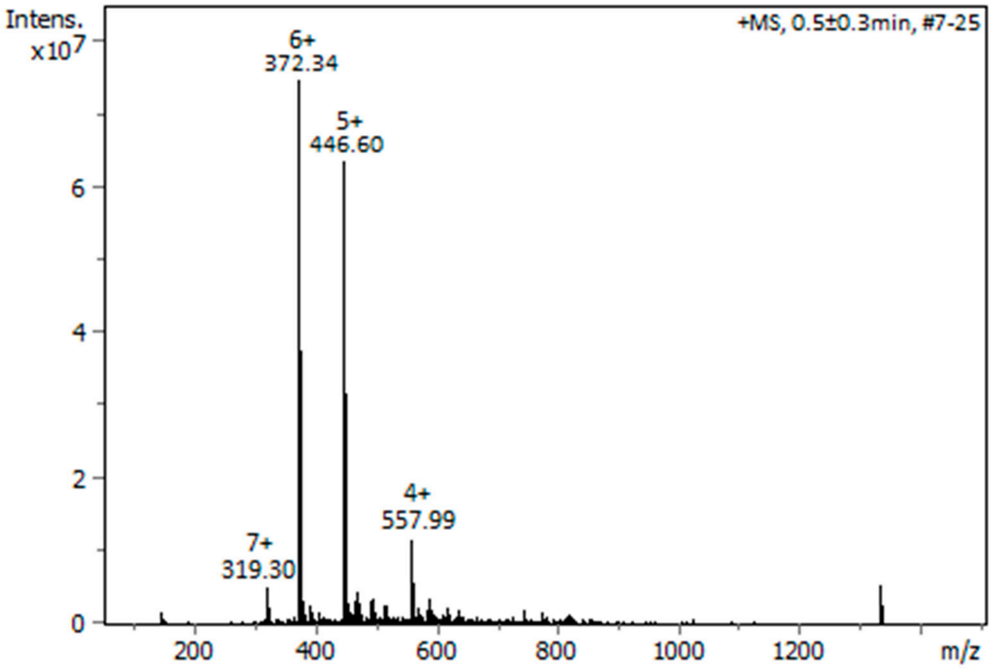

Figure S40. MS Spectrum of Dabcyl-Cys-(Arg)$_6$-Cys-(Gly)$_4$-Lys(Cf) orto BBMB. The identity of the peptide conjugate was determined using Bruker Amazon SL (Germany). The samples are dissolved in water-acetonitrile (50:50) with 0.1% formic acid.

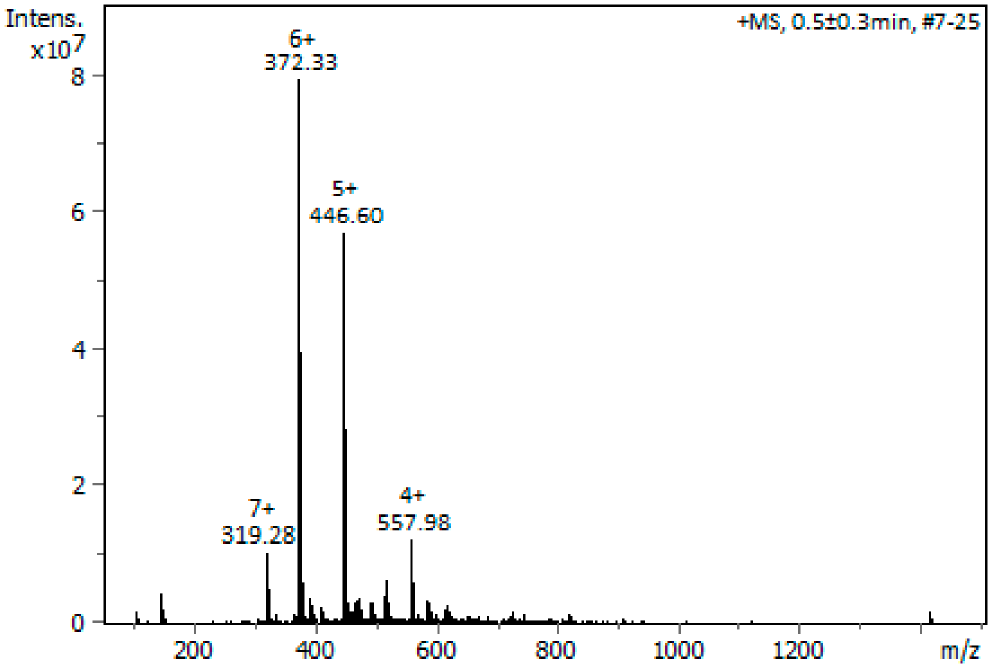

Figure S41. MS Spectrum of Dabcyl-Cys-(Arg)$_6$-Cys-(Gly)$_4$-Lys(Cf) para BBMB. The identity of the peptide conjugate was determined using Bruker Amazon SL (Germany). The samples are dissolved in water-acetonitrile (50:50) with 0.1% formic acid.

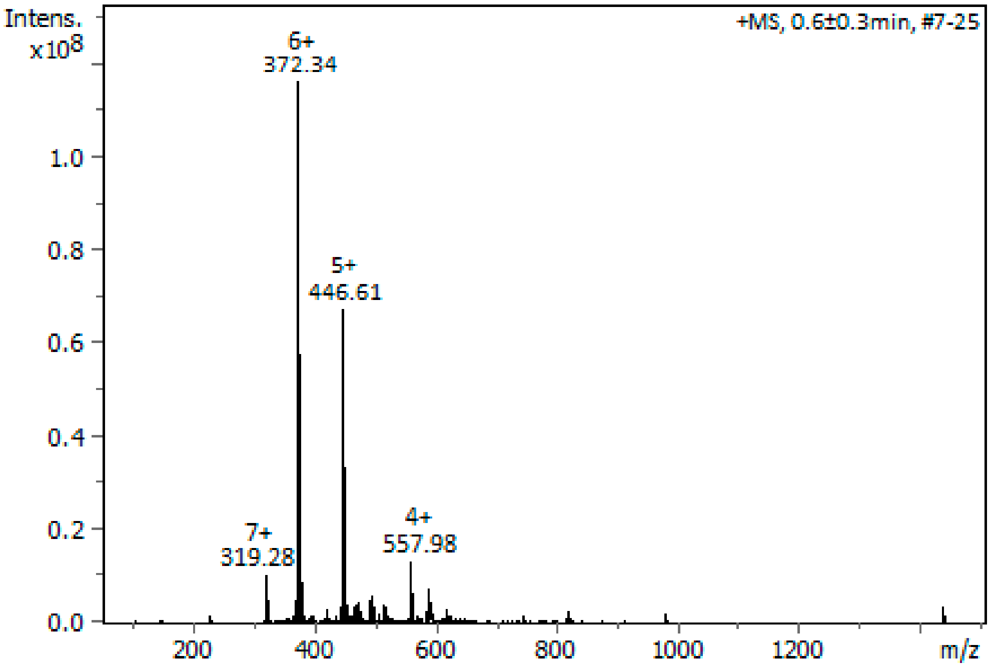

Figure S42. MS Spectrum of Dabcyl-(Arg)$_6$-Cys-(Gly)$_4$-(Cys)-Lys(Cf) orto BBMB. The identity of the peptide conjugate was determined using Bruker Amazon SL (Germany). The samples are dissolved in water-acetonitrile (50:50) with 0.1% formic acid.

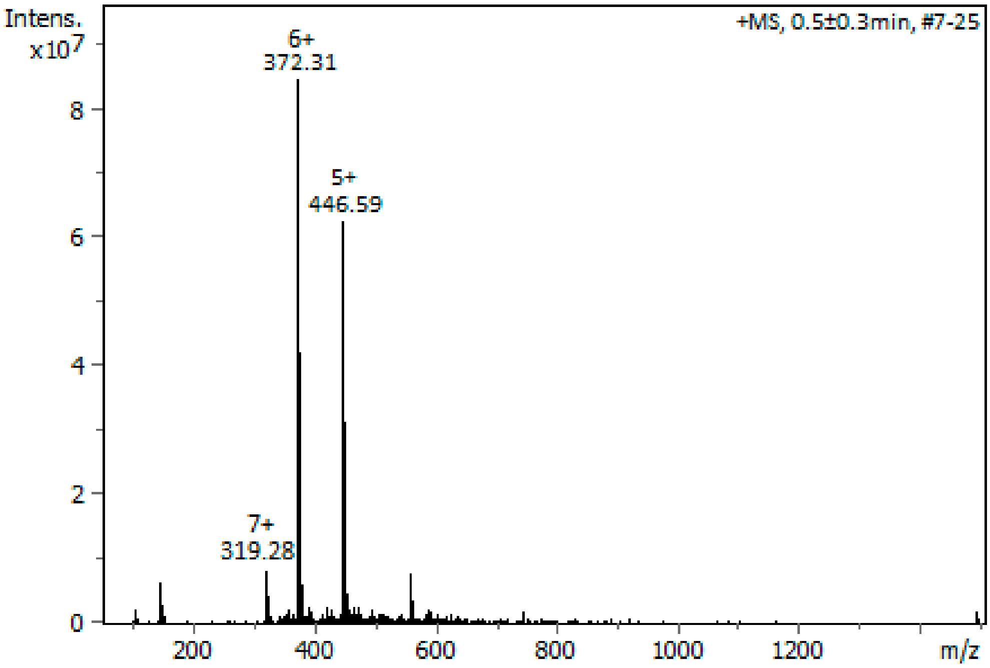

Figure S43. MS Spectrum of Dabcyl-(Arg)$_6$-Cys-(Gly)$_4$-(Cys)-Lys(Cf) para BBMB. The identity of the peptide conjugate was determined using Bruker Amazon SL (Germany). The samples are dissolved in water-acetonitrile (50:50) with 0.1% formic acid.

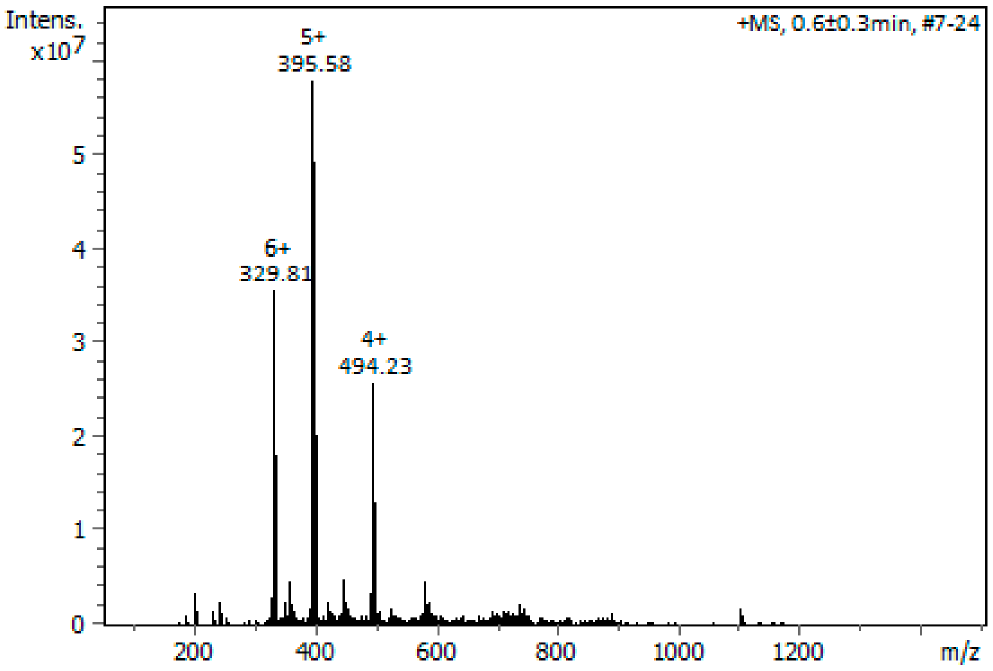

Figure S44. MS Spectrum of Acyl-Met-(Arg)$_6$-Met-(Gly)$_4$-Lys(Cf). The identity of the peptide conjugate was determined using Bruker Amazon SL (Germany). The samples are dissolved in water-acetonitrile (50:50) with 0.1% formic acid.

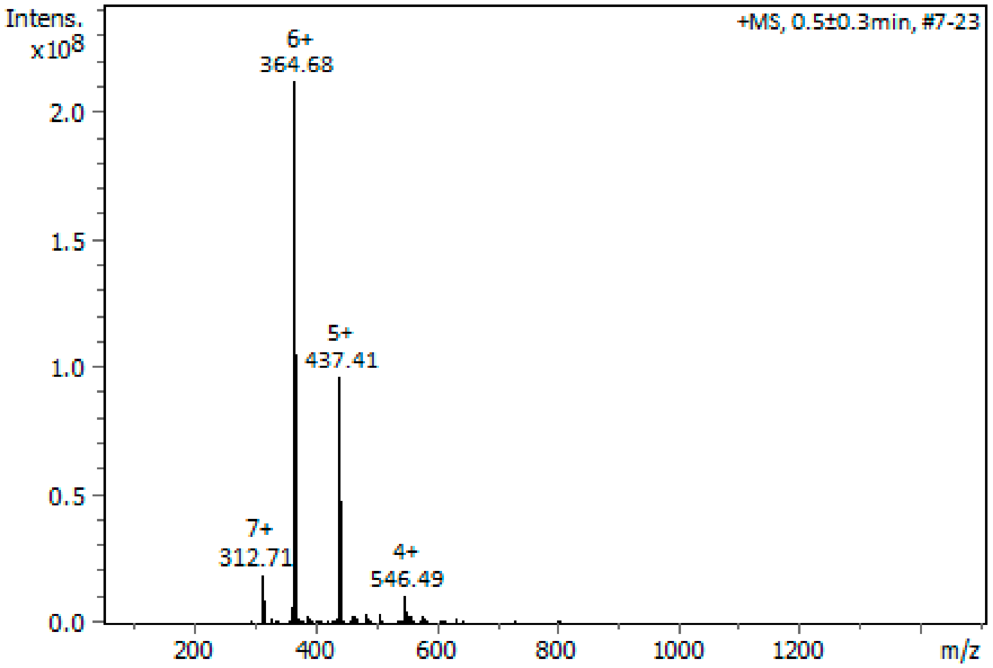

Figure S45. MS Spectrum of Dabcyl-Met-(Arg)$_6$-Met-(Gly)$_4$-Lys(Cf). The identity of the peptide conjugate was determined using Bruker Amazon SL (Germany). The samples are dissolved in water-acetonitrile (50:50) with 0.1% formic acid.

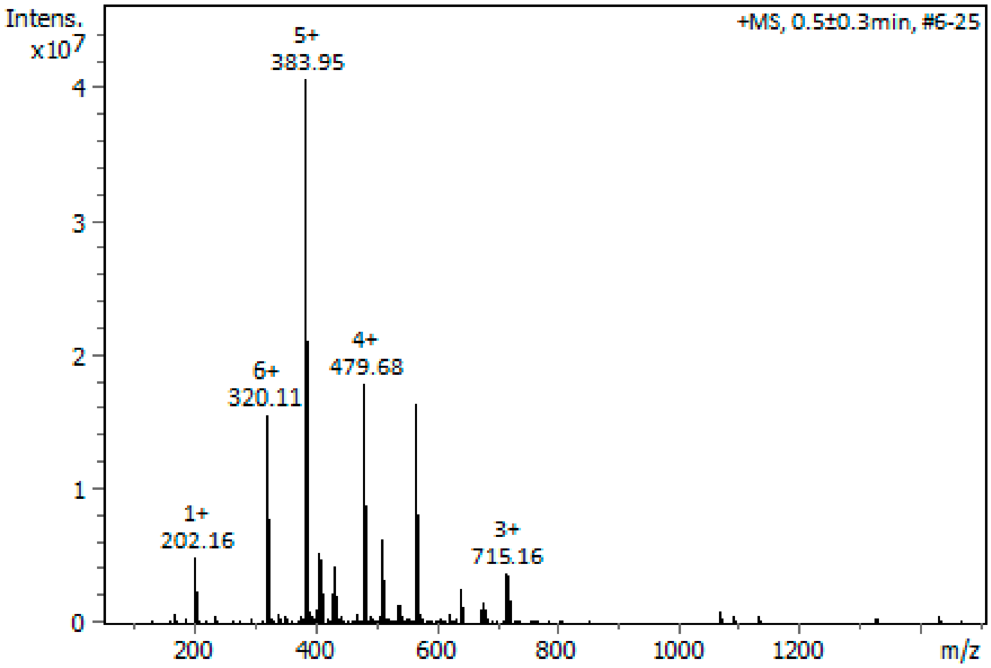

Figure S46. MS Spectrum of Acyl-Cys((Arg)$_6$-Cys)-(Gly)$_4$-Lys(Cf). The identity of the peptide conjugate was determined using Bruker Amazon SL (Germany). The samples are dissolved in water-acetonitrile (50:50) with 0.1% formic acid.

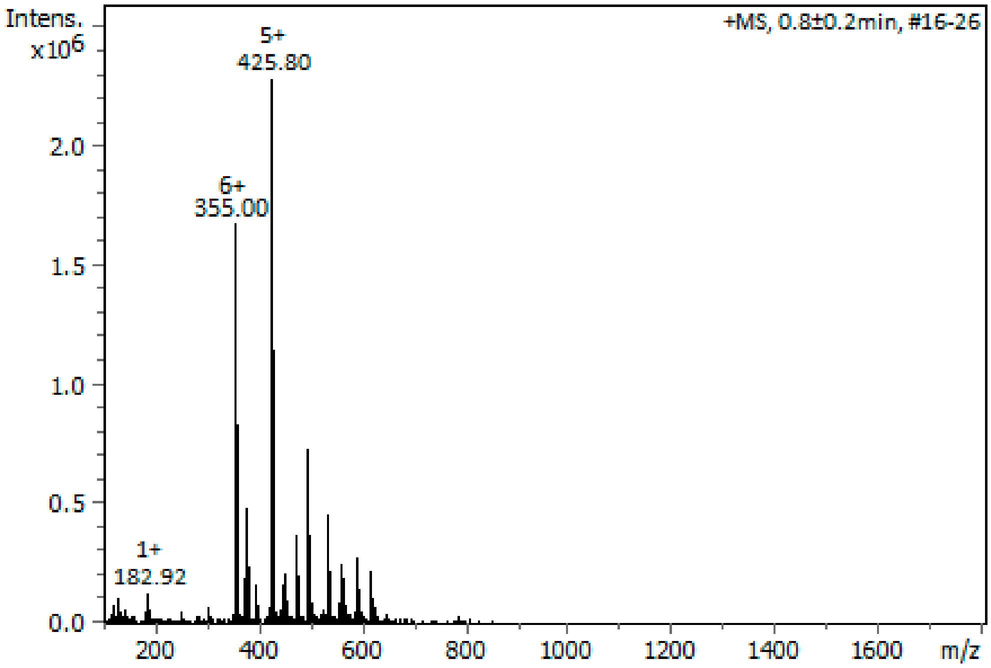

Figure S47. MS Spectrum of Acyl-Cys((Arg)$_6$-Cys)-(Gly)$_4$-Lys(Cf). The identity of the peptide conjugate was determined using Bruker Amazon SL (Germany). The samples are dissolved in water-acetonitrile (50:50) with 0.1% formic acid.

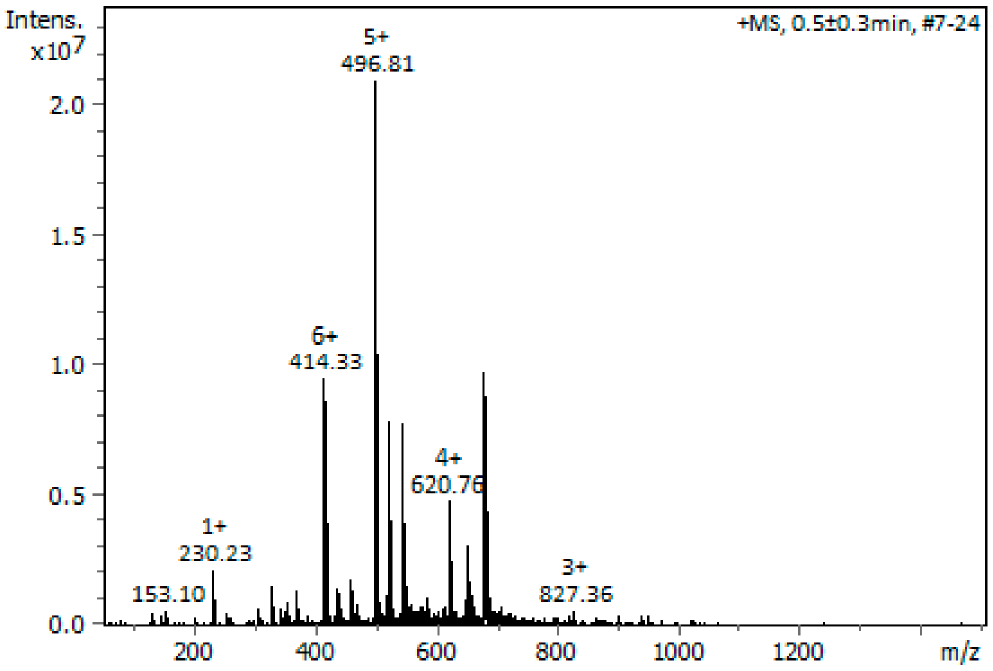

Figure S48. MS Spectrum of Dabcyl-Cys-(Arg)$_6$-Cys-(Gly)$_4$-Lys(DauSuc) orto BBMB. The identity of the peptide conjugate was determined using Bruker Amazon SL (Germany). The samples are dissolved in water-acetonitrile (50:50) with 0.1% formic acid.

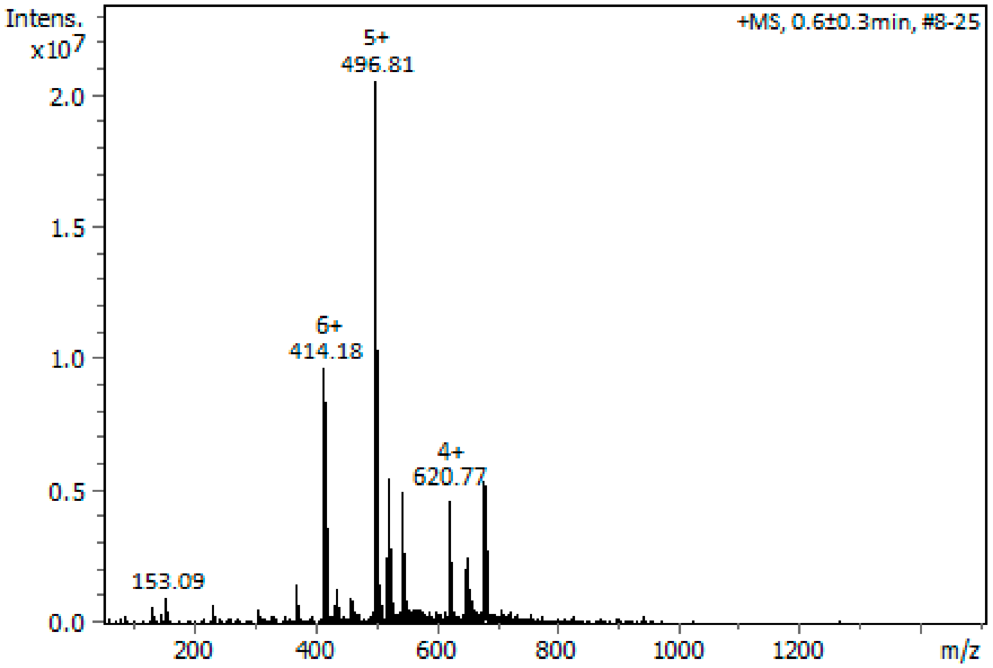

Figure S49. MS Spectrum of Dabcyl-Cys-(Arg)$_6$-Cys-(Gly)$_4$-Lys(DauSuc) para BBMB. The identity of the peptide conjugate was determined using Bruker Amazon SL (Germany). The samples are dissolved in water-acetonitrile (50:50) with 0.1% formic acid.

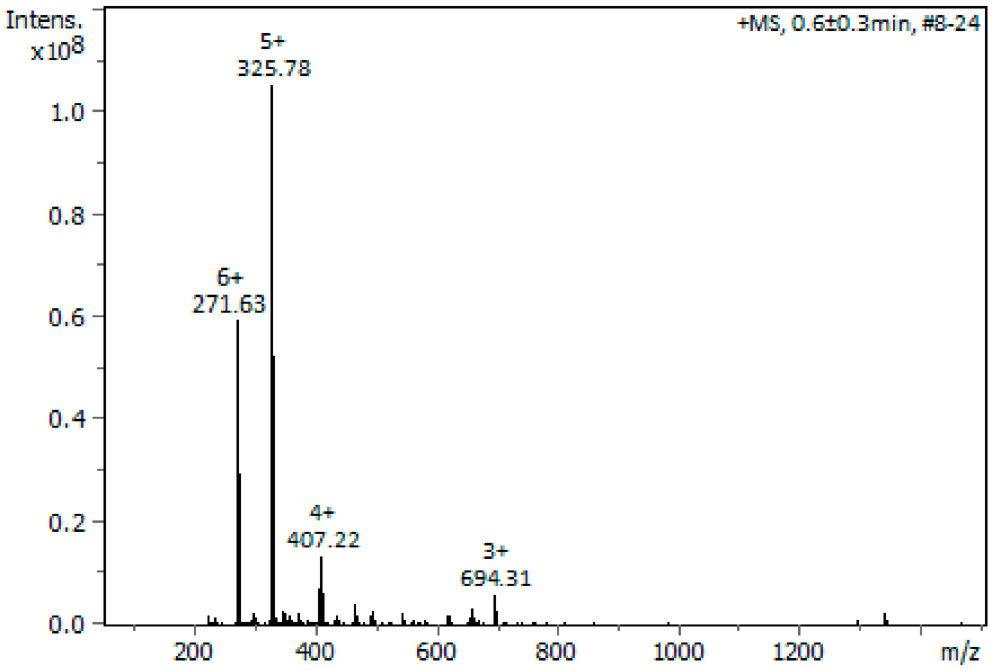

Figure S50. MS Spectrum of Cf-Arg$_8$. The identity of the peptide conjugate was determined using Bruker Amazon SL (Germany). The samples are dissolved in water-acetonitrile (50:50) with 0.1% formic acid.

2.3. In Vitro Cytostatic Effect of Conjugates

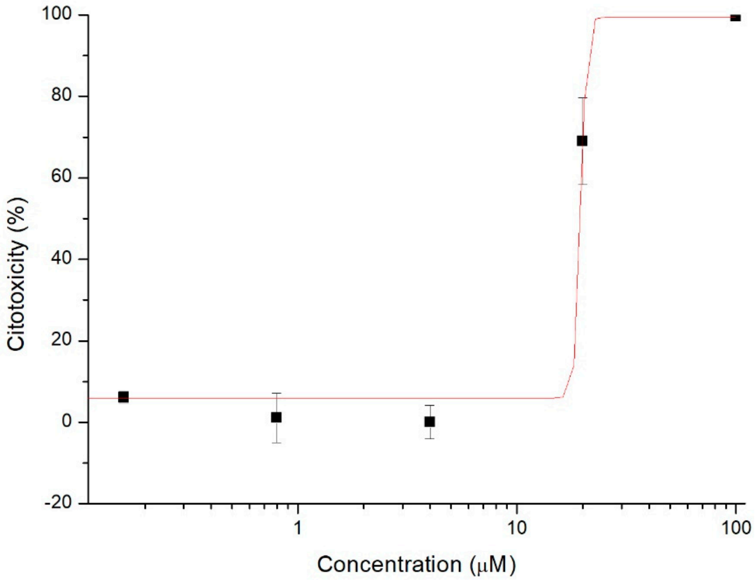

Figure S51. In vitro measurement on EBC-1 cells with Dabcyl-Cys-(Arg)$_6$-Cys-(Gly)$_4$-Lys(DauSuc) orto BBMB

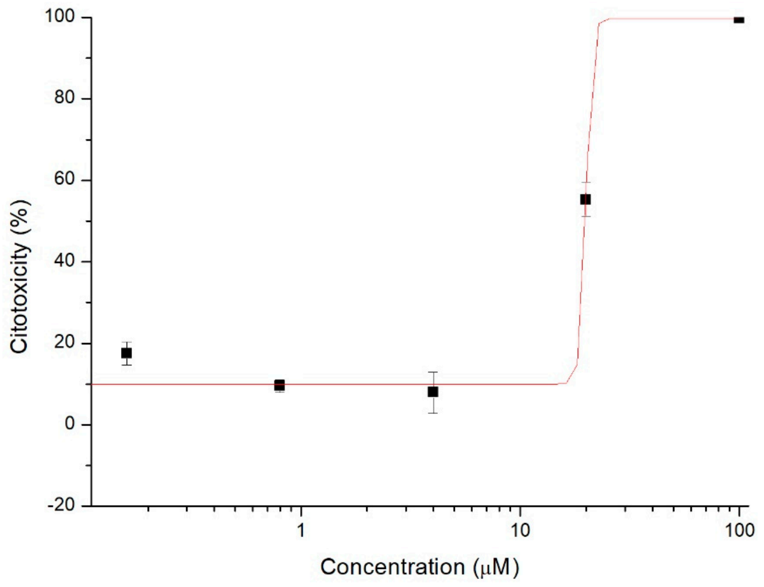

Figure S52. In vitro measurement on EBC-1 cells with Dabcyl-Cys-(Arg)$_6$-Cys-(Gly)$_4$-Lys(DauSuc) para BBMB

2.4. Stability measurement of linear and cyclic peptides

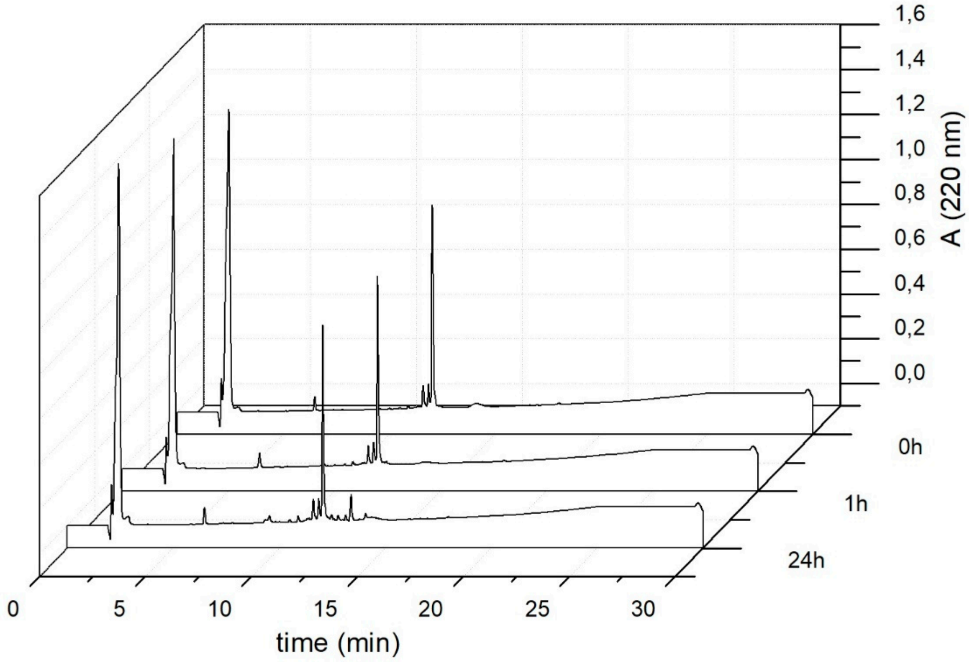

Figure S53. Stability measurement of Dabcyl-Cys-(Arg)$_6$-Cys-(Gly)$_4$-Lys(Cf) para BBMB. Samples were taken in 0, 1 and 24 hour time periods and measured with analytical HPLC.

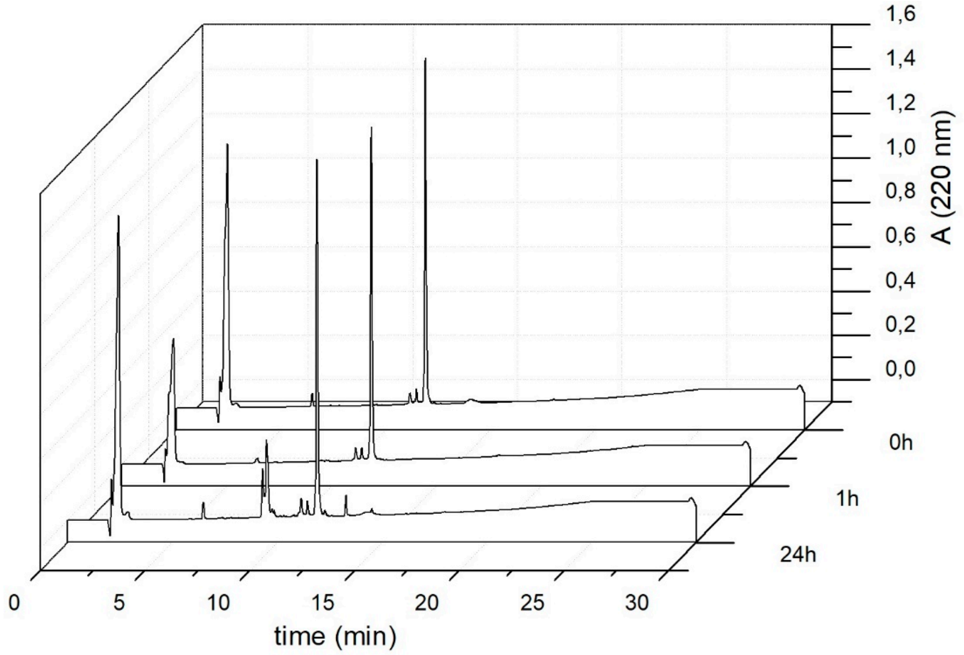

Figure S54. Stability measurement of Dabcyl-Cys-(Met)$_6$-Cys-(Met)$_4$-Lys(Cf). Samples were taken in 0, 1 and 24 hour time periods and measured with analytical HPLC.
